# Thymopentin treatment of murine premature ovarian failure via attenuation of immune cell activity and promotion of the BMP4/Smad9 signalling pathway

**DOI:** 10.7150/ijms.61975

**Published:** 2021-08-21

**Authors:** Xueqin Zhu, Jianjun Liu, Hao Pan, Zixiang Geng, William Huang, Te Liu, Bimeng Zhang

**Affiliations:** 1 Geriatrics Department, Punan hospital of Shanghai, Shanghai 200031, China.; 2 Trauma-Emergency & Critical Care Medicine Center, Shanghai Fifth People's Hospital, Fudan University, Shanghai 200240, China.; 3 College of Pharmacy, Chongqing Medical University, Chongqing 400016, China.; 4 Department of Acupuncture, Shanghai General Hospital, Shanghai Jiao Tong University School of Medicine, Shanghai 200086, China.; 5 Hainan Zhonghe Pharmaceutical Co., Ltd, Hainan, China.; 6 Shanghai Geriatric Institute of Chinese Medicine, Shanghai University of Traditional Chinese Medicine, Shanghai 200031, China.

**Keywords:** Premature ovarian failure, high-fat and high-sugar (HFHS), thymopentin (TP-5), BMP4/Smad9 signalling pathway, immune cell activity

## Abstract

Premature ovarian failure (POF) is a typical form of pathological aging with complex pathogenesis and no effective treatment. Meanwhile, recent studies have reported that a high-fat and high-sugar (HFHS) diet adversely affects ovarian function and ovum quality. Here, we investigated the therapeutic effect of thymopentin (TP-5) as a treatment for murine POF derived from HFHS and its target. Pathological examination and hormone assays confirmed that TP-5 significantly improved murine POF symptoms. And, TP-5 could reduce oxidative stress injury and blood lipids in the murine POF derived from HFHS. Flow cytometry and qPCR results suggested that TP-5 attenuated activation of CD3+ T cells and type I macrophages. RNA-Seq results indicated somedifferences in gene transcription between the TP-5 intervention group and the control group. KEGG analysis indicated that the expression of genes involved in the mTOR signaling pathway was the most significantly different between the two groups. Additionally, compared with the control groups, the expression levels of interleukin, NFκB, and TNF families of genes were significantly downregulated in the POF+TP-5 group, whereas expression of the TGFβ/Smad9 genes was significantly upregulated. Finally, immunofluorescence staining and qPCR confirmed that TP-5 promoted the polarization of Mø2 cells in the ovary by activating the expression of the BMP4/Smad9 signalling pathway. Thus, our study confirmed that TP-5 has a significant therapeutic effect on POF by upregulating BMP4/Smad9 signalling pathway so as to promote the balance and polarization of immune cell and reducing the release of inflammatory factors and reduce lipid oxidative stress injury.

## Introduction

Premature ovarian failure (POF) refers to a disorder in which women develop amenorrhea, infertility, low estrogen, and high gonadotropin levels, and lack of mature follicles before the age of 40. This condition is one of the most common causes of female infertility 1-5. The development of POF is closely related to the condition and quality of ovarian granulosa cells (OGCs) 1-5. Aging and apoptosis of OGCs are important causes of decreased ovarian reserve function 1-5. It has been reported that inflammation can induce significant necrosis or apoptosis of OGCs, resulting in loss of their original function and ultimately inducing POF 1-5; however, the specific mechanism by which this occurs is still unclear, and there are currently no effective treatment for POF. Previously, we demonstrated that oxidative stress-induced injury adversely affects OGC health 6. Recent studies have reported that a high-fat and high-sugar (HFHS) diet increases the risk of obesity, tumor formation, and cardiovascular disease 7-9 and adversely affects ovarian function and ovum quality 10-12. Meanwhile, we have reported that HFHS activated the Dab2ip/Ask1/p38-Mapk signaling pathway and promoted γH2A.X phosphorylation by inhibiting the expression of endogenous miR-146b-5p, which resulted in OGC aging and POF development 13. Therefore, above evidences indicated that HFHS diet-induced obesity was closely related to the occurrence of POF.

Thymosin II is a single 49-amino acid polypeptide isolated from the thymus 14-18. Thymopentin (TP-5), which is an active component of thymosin II, which is secreted by the thymus and has the same physiological functions 14-18. TP-5 is composed of five amino acids: arginine, lysine, aspartic acid, valine, and tyrosine with the molecular formula C_30_H_49_N_9_O_9_ and a molecular weight of 679.77. Studies have shown that TP-5 has significant immunomodulatory effects 14-18. It can promote the differentiation of T cells by increasing cAMP and intracellular GMP levels by binding to T cell-specific receptors, thereby inducing a series of intracellular reactions and regulating immune functions. In addition, TP-5 induces T cell differentiation, promoting the development, maturation, and activation of T lymphocytes subsets, and regulating the CD4/CD8 homeostasis of these subsets 14-18. TP-5 has significant effects on the re-establishment of immune function after chemoradiotherapy in patients with malignant cancers, and in the immune system of elderly and immunodeficient individuals 14-18. In addition, TP-5 has therapeutic effects on autoimmune diseases such as rheumatoid arthritis, lupus erythematosus, type II diabetes, and female climacteric syndrome 14-18. However, the use of TP-5 to treat POF has not yet been reported.

Based on existing evidence, we investigated the therapeutic effects of TP-5 intervention in a murine POF derived from HFHS and explored the underlying mechanism from the perspective of immune cell activity and the release of inflammatory factors and lipid oxidative stress injury.

## Materials and methods

### POF model and TP-5 treatment

Thirty female C57BL/6 mice (aged 10 weeks) were purchased from the Experimental Animal Center of Shanghai University of Traditional Chinese Medicine (China). According to our previously established protocol 5, 7, 13, the mice were divided into three groups: a group untreated with any reagents; a negative control group treated with saline daily by intraperitoneal injectionand HFHS (high-fat diet (8 g/kg bodyweight) and administered 200 μL of 30% high fructose syrup once a day via gavage); an experimental group treated with 5 mg/kg TP-5 (Hainan Zhonghe Pharmaceutical Co., Ltd, Hainan, China)daily by intraperitoneal injection and HFHS (high-fat diet (8 g/kg bodyweight) and administered 200 μL of 30% high fructose syrup once a day via gavage). Following treatment, experiments using the animal model were conducted within 2 months. The mice were anesthetized and euthanized. Ketamine (10 mg/mL)/Xylazine (10 mg/mL) was used for mouse anesthesia and administered 10 μL/g by intraperitoneal injection. All the animal experiments were conducted in accordance with the guidelines of the National Institutes for Health for the care and use of laboratory animals. The study protocol was also approved by the Committee on the Use of Live Animals in Teaching and Research, Experimental Animal Center of Shanghai University of Traditional Chinese Medicine, Shanghai, China (No. Shage20180001884).

### Hematoxylin and eosin (HE) staining

According to a previously reported method 4, tissues were fixed in 4% paraformaldehyde at room temperature for 12 h. Frozen tissue sections were prepared (thickness approximately 5 μm) and fixed in 95% anhydrous ethanol for 2 min. Sections were then stained in hematoxylin for 5 min, and differentiated in differentiation solution for 2 min. Sections were immersed in weak ammonia solution for 3 min, washed with deionized water for 5 min, and then stained with eosin for 5 min. After washing with deionized water for 5 min, tissue sections were immersed in 70%, 80%, and 90% alcohol solution once for 1 min, washed with anhydrous ethanol twice (1 min per wash), cleared in xylene twice (1 min per wash), and mounted using neutral balsam. These reagents and materials were all purchased from Beyotime Biotechnology Co., Ltd., (Zhejiang, China).

### Immunofluorescence staining and flow cytometry

According to previously reported methods 5, cell samples were fixed in 1 ml 4% paraformaldehyde (Sigma-Aldrich) at room temperature for 30 min and blocked in blocking solution (Beyotime Biotechnology, Hangzhou, China) at 37 °C for 60 min. The blocking solution was discarded, and the cells were washed three times (5 min per wash) with an immunohistochemistry washing solution (Beyotime Biotechnology) at room temperature. Samples were then incubated at 37 °C for 45 min with primary detection antibodies (anti-mouse CD3 monoclonal antibody (mAb) (17A2), PE (Cat #12-0032-82), anti-mouse CD4 mAb (45-0042-82), PerCP-Cyanine5.5 (Cat #45-0042-82), anti-mouse CD8a mAb (53-6.7), PerCP-Cyanine5.5 (Cat #45-0081-82), anti-mouse F4/80 mAb (BM8), Alexa Fluor 488 (Cat #53-4801-82), anti-mouse CD68 mAb (FA-11), PE, (Cat #12-0681-82), anti-mouse CD206 (MMR) mAb (MR6F3), PE (Cat #12-2061-82); all from eBioscience, MA, USA; rabbit anti-mouse TGF-β antibody (Cat #3711), rabbit anti-mouse BMP4 (6B7) antibody (Cat #4680), Smad 1/5/9 Antibody Sampler Kit (Cat #12656), all from Cell Signaling Technology, MA, USA and Abcam, MA, USA). The antibodies were discarded, and the cells were washed three times (5 min per wash) with the immunohistochemistry washing solution (Beyotime Biotechnology) at room temperature. Samples were then incubated at 37 °C for 45 min with secondary detection antibodies (goat anti-rabbit IgG H&L (Alexa Fluor® 647) (ab150083); Abcam, MA, USA). The antibodies were discarded, and the cells were washed three times (5 min per wash) with the immunohistochemistry washing solution (Beyotime Biotechnology) at room temperature. Finally, the cells were mounted in immunofluorescence mounting fluid (Sigma-Aldrich). The cells were then detected using a flow cytometer (Cytomics FC500, BECKMAN).

### Establishment of cDNA sequencing libraries and high-throughput RNA-Seq

The following analysis was conducted by Kang Chen Bio-tech (Shanghai, China). According to their experimental procedures, a random fragment sequencing library was constructed using a SOLiD Whole Transcriptome Analysis Kit (Life technologies, MA, USA). Nucleic acid cleaving reagents were added, and the mRNA was randomly disrupted into short segments in a shaking incubator. First-strand cDNA was reverse-transcribed using the fragmented mRNA as the template. Second-strand cDNA was synthesized using a second-strand DNA synthesis reaction system consisting of DNA polymerase I, dNTPs and RNase H (Sigma-Aldrich). The synthesized DNA was purified and recovered using a DNA purification kit. An adenine (A) base was added to the 3' end of the cDNA, followed by ligation to the adapter, to complete the blunt end repair reaction. Subsequently, DNA fragment size selection was performed. Finally, the cDNA was used for PCR amplification to obtain a sequencing library. The quality of the constructed library was evaluated using an Agilent 2100 Bioanalyzer and the ABI StepOnePlus Real-Time PCR System and was subjected to high-throughput sequencing using an Illumina HiSeq ™ 2000 Sequencer after passing quality control.

### ELISA

The plasma levels of mouse estradiol (E_2_) were determined using an ELISA kit (Westang Bio, Shanghai, China) according to the manufacturer's instructions. Briefly, 100 μl of mouse E_2_ standardized to 8,000, 4,000, 2,000, 1,000, 500, 250 and 125 pg/mL or diluted mouse plasma were added to anti-E_2_ antibody-precoated microwells and incubated for 60 min. After three washing steps, HRP-conjugated detection antibodies were added, followed by the substrate solution. The absorbance was measured at 450 nm.

The plasma levels of mouse IL-1β, IL-6, IL-22, TNFα and IFNγ were determined using ELISA kits (Westang Bio, Shanghai, China) according to the manufacturer's instructions. Briefly, 100 μl of mouse IL-1β, IL-6, or TNFα standardized to 1,000, 500, 250, 125, 60, 30, 15, 0 ng/ml or diluted mouse plasma were added to microwells pre-coated with antibodies for the detection of the listed cytokines and incubated for 60 min. In addition, 100 μl of mouse IL-22, or IFNγ standardized to 1,000, 500, 250, 125, 60, 30, 15, 0 pg/ml or diluted mouse plasma were added to microwells pre-coated with antibodies for the detection of the listed cytokines and incubated for 60 min. After three washing steps, HRP-conjugated detection antibodies were added, followed by the substrate solution. The absorbance was measured at 450 nm.

### ATP assay

The ATP assay was performed using an enhanced ATP test kit (Beyotime), according to the manufacturer's instructions. For the ATP assay, 1×10^5^ cells/mL were lysed thoroughly with 200 µL of sample lysate, centrifuged at 12000 *g* for 5 min at 4 °C. The supernatant was carefully collected. To prepare the ATP standard curve, the ATP standard was adjusted with a dilution buffer to final concentrations of 0.01, 0.03, 0.1, 0.3, 1, 3, and 10 μM, and this curve was used as a reference to determine the value of the sample. Working solutions for ATP detection were freshly made in accordance with the kit's requirements. For the assay, 100 μL of working solution was added to the test and sample wells at the same time, and the plates were incubated at room temperature for 5 min. Then, 20 μL of sample or standard was added to the wells at the same time, quickly mixed, incubated at room temperature for 5 s, and the RLU value was measured on a luminometer.

### SOD assay

According to the instructions of the SOD activity test kit (Beyotime), briefly, 1×10^5^/mL of cells were lysed thoroughly using with 200 µL of sample lysate, centrifuged at 12000 *g* for 5 min at 4 °C, and the supernatant was collected. Then, fresh WST-8 enzyme working solution was prepared by mixing 151 µL of SOD detection buffer with 8 μL of WST-8 and 1 μL enzyme solution. At the same time, the SOD standard was diluted to 100, 50, 20, 10, 5, 2, 1 U/mL to obtain a gradient. Then, 20 μL of the cell lysis supernatant or standard solution wasadded to 160 μL of fresh WST-8 enzyme working solution and 20 μL reaction starting solution, mixed well, and incubated at 37 °C for 30 min. Absorbance was measured at 450 nm.

### RNA extraction and qPCR

Total RNA was extracted from each group of cells in accordance with the instructions of Trizol Reagent (Invitrogen) and treated with Dnase I (Sigma-Aldrich) to remove residual genomic DNA. cDNA synthesis was carried out using ReverTra Ace-α First-Strand cDNA Synthesis Kit (Toyobo). qPCR analysis was conducted using a RealPlex4 real-time PCR detection system (Eppendorf Co. Ltd., Germany), with SYBR Green real-time PCR Master Mix (Toyobo) as a fluorescent dye for nucleic acid amplification.The following reaction conditions were used for 40 cycles: denaturation at 95 °C for 15 s, annealing at 58 °C for 45 s,andelongationat 72 °C for 42 s. For each sample, the maker gene Ct values were normalized with the following formula: ΔCt =Ct_genes-Ct_18sRNA, and ΔΔCt = ΔCt_all_groups-ΔCt_blankcontrol_group. The mRNA levels were calibrated according to the 18S rRNA levels.

### Western blot

Briefly, total protein was extracted from cells in each group. Protein content was determined by using the BCA assay (Pierce Biotechnology, Inc., Rockford, IL, USA). Briefly, 20 μg protein samples were electrophoresed on 12% SDS-PAGE. These parated proteins were transferred to a polyvinylidene difluoride (PVDF) membrane (Millipore, Billerica, MA, USA) for 45 min at 37 °C after the blocking and membrane washing 4 times with TBST for 1 mineach time. Each membrane was washed and incubated with the secondary antibodies for 45min. Immunoreactivity was visualized by performing an enhanced chemiluminescence (ECL) assay using an ECL kit from Perkin-Elmer LifeScience (Norwalk, USA).

### Statistical analysis

Each experiment was performed as least three times, and data represent the mean ± SD where applicable. Differences were evaluated using Student's *t*-test. *P*< 0.05 was considered to indicate statistical significance. In addition, about the follicular count method, under the microscope low power field of vision, the total number of normal follicles (including primordial follicles, secondary follicles, sinus follicles, etc.) and atresia follicles in a complete ovarian tissue were calculated respectively. Then, according to the formula (total number of atresia follicles/total number of normal follicles) ×100%, the proportion of atresia follicles can be obtained.

## Results

### TP-5 significantly improveslipidoxidative stress injury and POF symptoms

**Figure 1:**
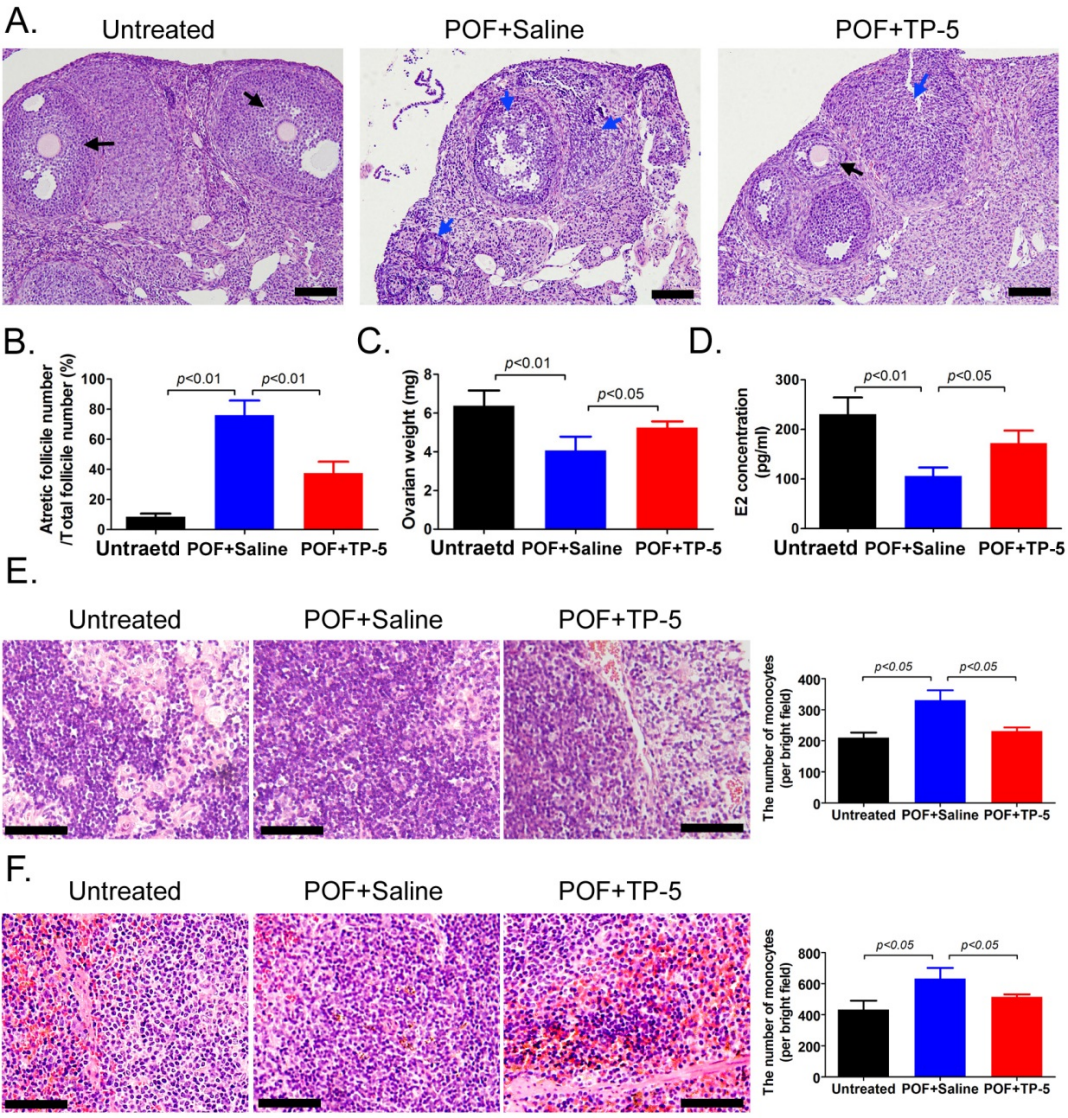
** TP-5 significantly improves POF symptoms. (A)** H&E staining of each group of ovarian samples. Black arrow indicates normal follicle, 400× magnification; blue arrow indicates atretic follicle. Scale bars: 30 µm. **(B)** Calculation of atretic follicle proportions. **(C)** Determination of ovarian weight. **(D)** ELISA of estrogen levels in peripheral blood. **(E)** H&E staining of thymus tissue in each group, 200× magnification. Scale bars: 30 µm.** (F)** H&E staining of spleen tissue in each group, 200× magnification. Scale bars: 30 µm.

**Figure 2:**
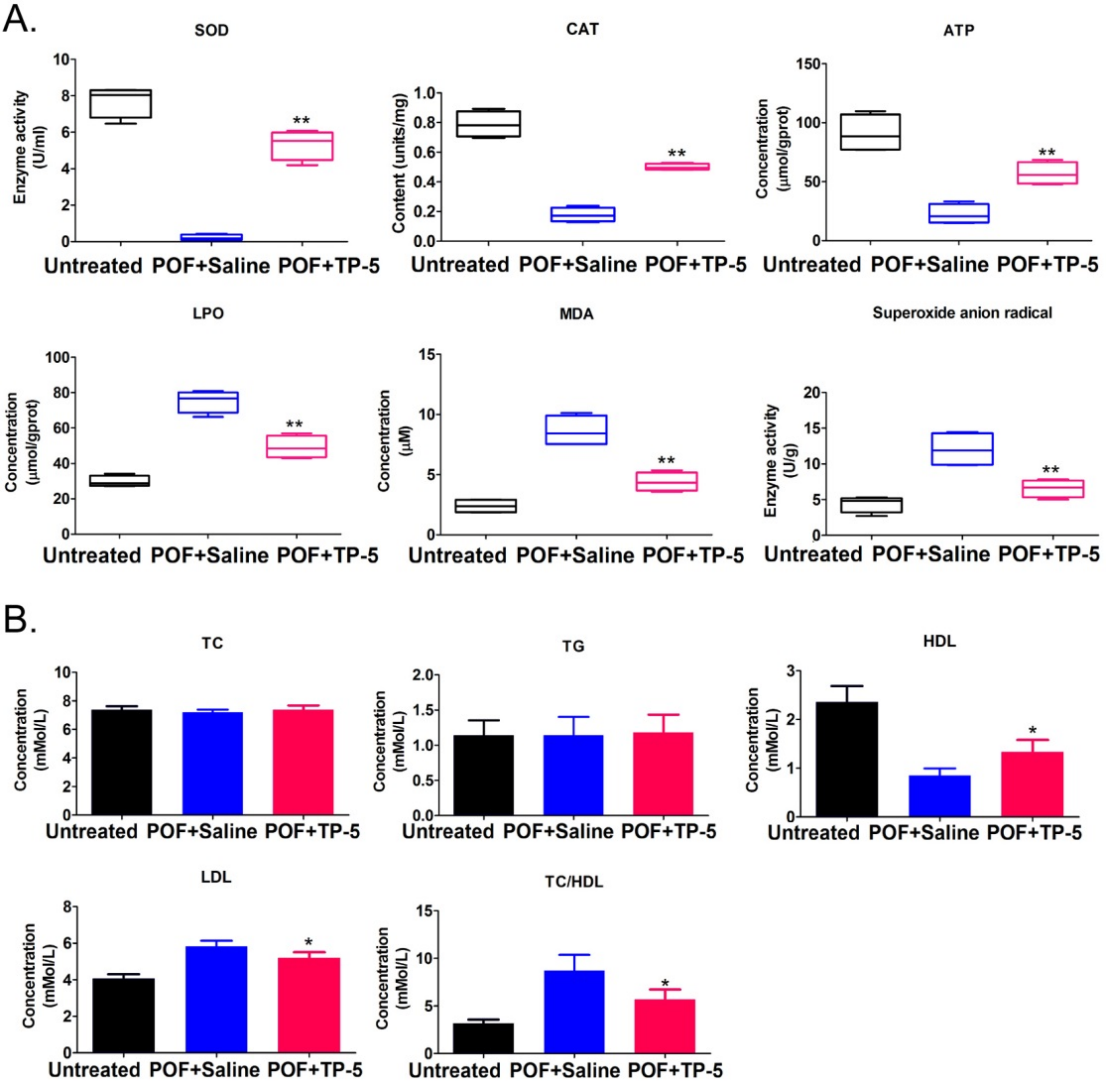
** The results of oxidative stress and blood lipid biomarkers. (A)** Oxidative stress biomarkers assay. **p<0.01 vs POF+Saline; t test; n=4.SOD, Superoxide dismutase; CAT, Catalase; ATP, Adenosine 5'-triphosphate; LPO, Lipid peroxidation; MDA, Malondialdehyde. **(B)** Blood lipid biomarkers assay. *p<0.05 vs POF+Saline; t test; n=4.

Histopathological examination of ovarian tissue from each group showed few follicles in the ovarian tissue of the POF+Saline (HFHS+Saline) mice group, whereas multiple mature oocytes and follicular tissues were found in the ovaries of the normal (Untreated) group (Figure 1A). In TP-5-treated mice (HFHS+TP-5), the phenomenon of atretic follicles in ovarian tissue was significantly improved. Follicle counting indicated that the proportion of atretic follicles in the ovaries from the POF group was significantly higher than that from the Untreated group, whereas the proportion of atretic follicles in the ovaries from the TP-5 treated group was significantly lower than that from the POF group (Figure 1A, B). In addition, the weights of the POF mouse ovaries were significantly lower than those of the untreated group, whereas the weights of the ovaries of TP-5-treated mice were significantly higher than those of the POF group (Figure 1C). Peripheral blood E_2_ levels were significantly lower in POF mice than those in the untreated group, whereas peripheral blood E_2_ levels were significantly higher in the TP-5-treated group than those in the POF group (Figure 1D). Meanwhile, pathological evaluation showed that HFHS intervention in mice resulted in an increase in monocytes in the spleen and thymus tissues, which was alleviated by TP-5 intervention (Figure 1E, F). Besides, compared with ovarian tissues in murine POF+Saline group, CAT, ATP contents and SOD activity were higher and LPO, MDA, Superoxide anion radical contents were significantly lower in the TP-5-treated groups (Figure 2A). And, the levels of high density lipoprotein (HDL) in peripheral blood from TP-5 treated mice was significantly higher than it in POF+Saline group (Figure 2B). But, the levels of lowdensity lipoprotein (LDL) and the ratio of total cholesterol (TC) to HDL in peripheral blood from TP-5 treated mice were significantly lower than them in POF+Saline group (Figure 2B).These experimental results suggested that TP-5 significantly improved POF symptoms and HFHS mediated lipid oxidative stress injury.

### TP-5 significantly improves immune cell proportion and activity

**Figure 3:**
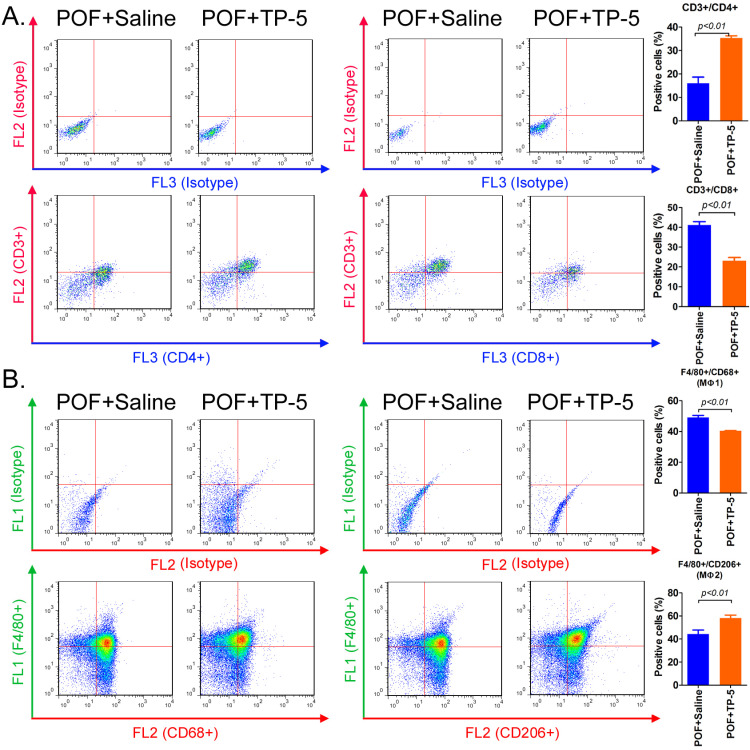
** Flow cytometric analysis of immune cell proportions. (A)** T lymphocytes. **(B)** Macrophages.

Flow cytometric analysis of the type and number of immune cells in the ovaries in each group of mice showed that the proportion of helper T cells (CD3+/CD4+) in the ovarian tissue from the TP-5 intervention group was significantly higher than that in the tissues from the control group. In contrast, the proportion of activated T cells (CD3+/CD8+) in the ovarian tissue from the TP-5 intervention group was significantly lower than that in the tissues from the control group (Figure 3A). Simultaneously, the proportion of macrophages (Mφ1) cells (F4/80+/CD68+) in the ovarian tissue of the TP-5 intervention group was significantly lower than that in the control group, whereas the proportion of Mφ2 cells (F4/80+/CD206+) was significantly higher than that in the control group (Figure 3B). In addition, the results of qPCR revealed that the expression levles of other inflammatory factor activation biomarkers mRNAs, such as Il1b, Il6, Ifng in CD3+ T cells in POF+TP-5 treated group were significantly lower than those in the control group, exception to Il2 (Figure S1). Meanwhile, the results of qPCR also revealed that the expression levles of other inflammatory factor activation biomarkers mRNAs, such as Il1b, Tnf, Ifng in F4/80+ macrophages (Mφ) in POF+TP-5 treated group were significantly lower than those in the control group, exception to differentiation promoting factor Tgfb1 (Figure S1). These experimental results indicated that TP-5 attenuated proportion and activation of CD3+ T cells and type I macrophages.

### TP-5 alters the expression of multiple genes and associated signalling pathways in ovarian tissue

**Figure 4:**
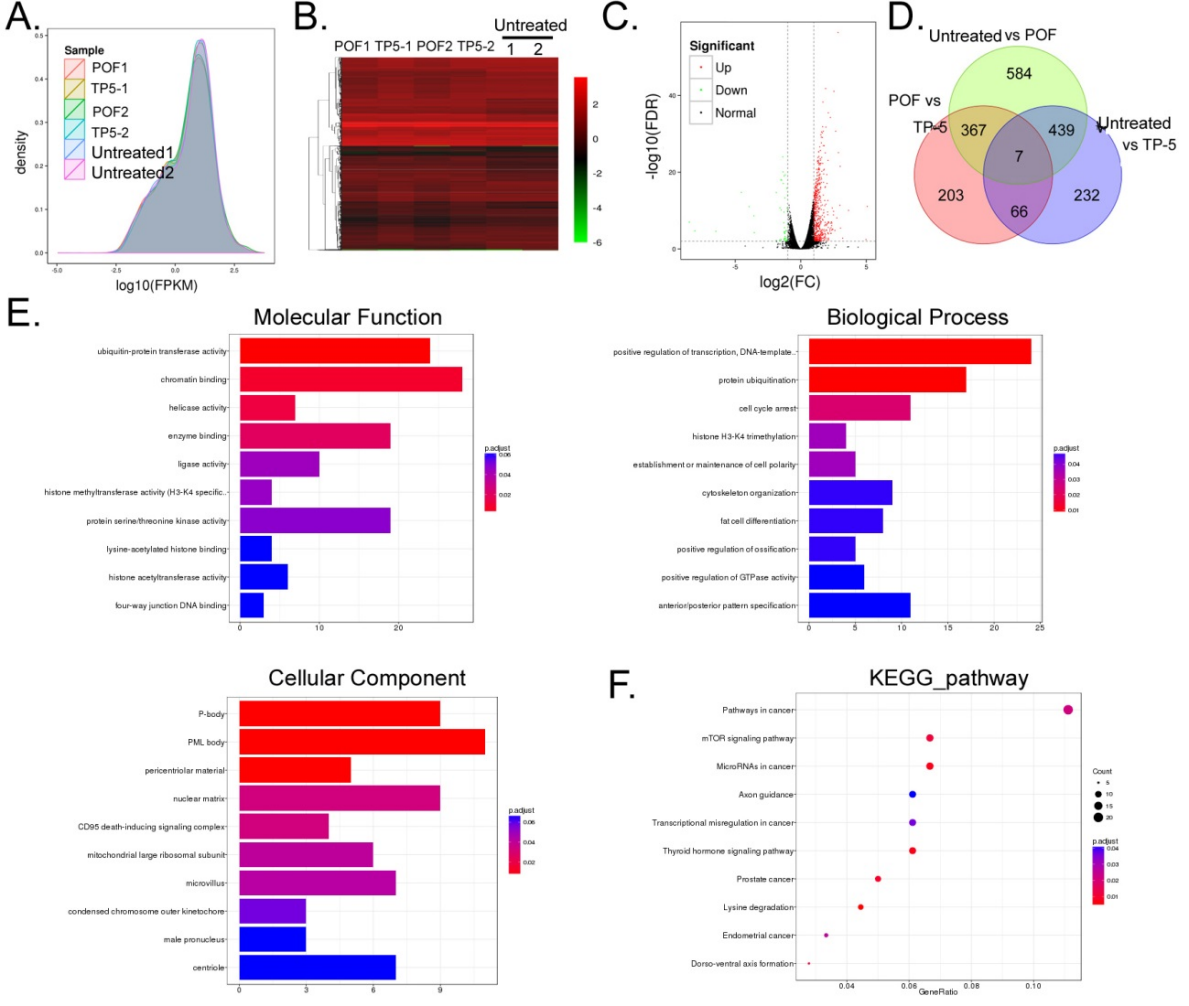
** RNA-Seq analysis of gene expression profiles of ovarian tissue in each group. (A)** Distribution density of differentially expressed genes in each group. **(B)** Gene expression heatmap for each group of samples; green and red colors represent downregulated and upregulated expression, respectively. **(C)** Volcano plot analysis of gene expression for each group of samples; green and red dots represent downregulated and upregulated expression, respectively. **(D)** Analysis of similarities and differences between gene expression profiles for each group. **(E)** GO analysis showed that the differences in gene expression between the TP-5 and POF groups were most significant in the cellular process, single-organism process, cell part, binding, and catalytic activity. **(F)** KEGG analysis showed that genes involved in mTOR signalling pathway.

Ovarian tissue mRNA was collected from a total of six samples in the POF+TP-5, POF+Saline and Untreated groups and sequenced using RNA-Seq transcriptome sequencing. A total of 52.47 Gb of clean data were obtained. The clean data for each sample reached 8.74 Gb, and the minimum Q30 base percentage was 93.32%. The clean reads for each sample were aligned with the designated reference genome sequence, with efficiency ranging from 95.15% to 96.63%. RNA-Seq analysis showed differential expression of 14,448 genes among the three groups (Figure 4A, B, C, Table S1). Of these, the POF+TP-5 group had a significant change in mRNA expression levels for a total of 50 genes compared with the POF+Saline group, of which 43 were significantly upregulated (POF+TP-5/POF+Saline>10 and *P* < 0.01) and 17 were significantly downregulated (POF+TP-5/POF+Saline>0.1 and *P* < 0.01). The number of upregulated genes was significantly higher than the number of downregulated genes (Figure 3D, Table S1). Pairwise alignment of the RNA-Seq results revealed that seven genes were differentially expressed in every group.

GO analysis showed that two biological processes, cellular and single-organism processes, contained the genes with the most significant differences in expression between the POF+TP-5 and POF+Saline groups (Figure 4E). Regarding organelle composition, cells and their organelles contained the genes with the most significant differences in expression (Figure 4E). With regard to protein molecular function, binding and catalytic activity pathways contained the functional genes with the most significant differences in expression (Figure 4E). Finally, with respect to the Kyoto Encyclopedia of Genes (KEGG), mTOR signalling pathway contained the genes with the most significant differences in expression (Figure 4F). These experimental results indicated that TP-5 alters the expression of multiple genes and their associated signalling pathways in ovarian tissue.

### TP-5 significantly alters the expression level of immunoregulatory factors

**Figure 5:**
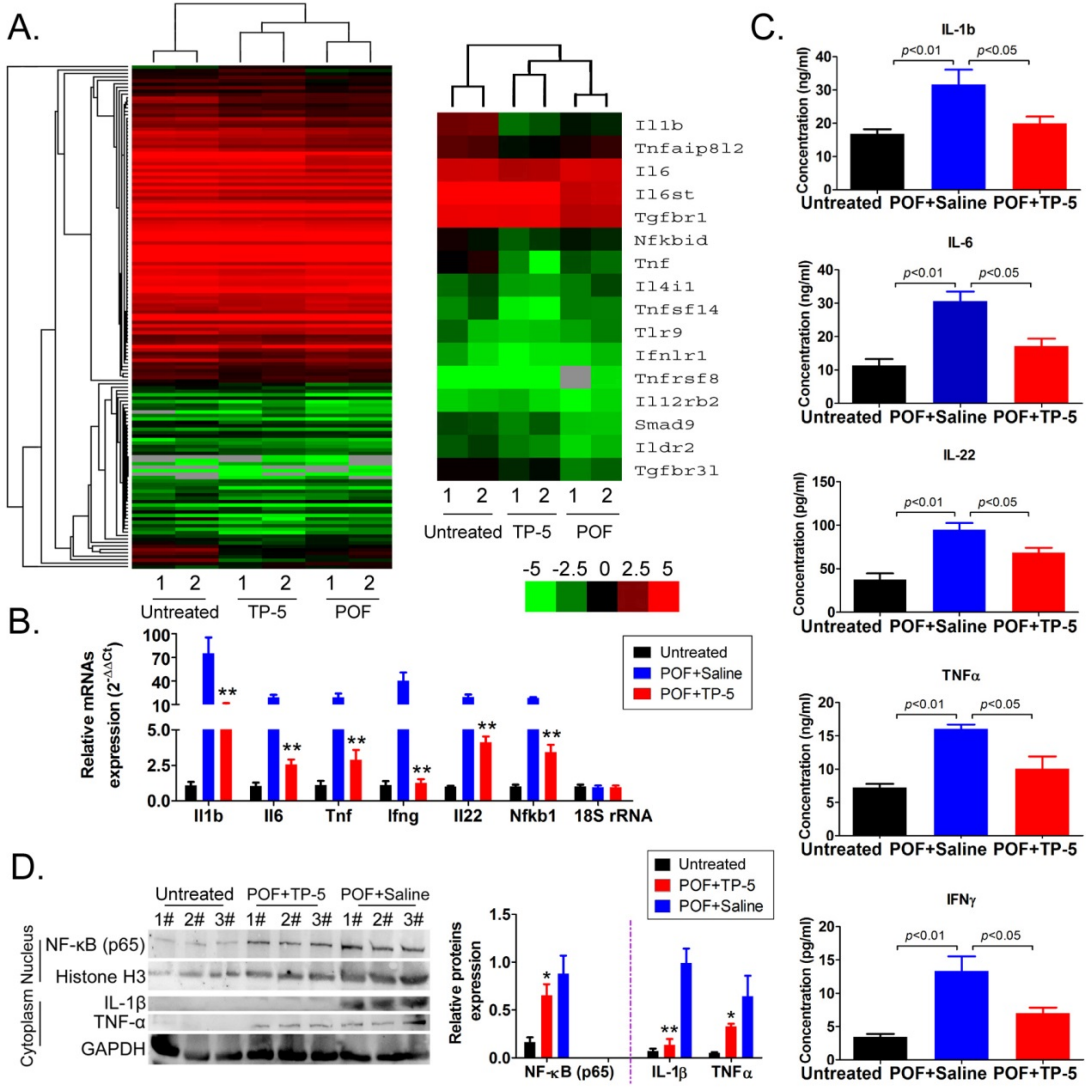
**RNA-Seq analysis of differentially expressed genes of immunoregulatory factors. (A)** Heatmap analysis of differential expression of genes in the interleukin, NFκB, and TNF families; green and red colors represent downregulated and upregulated expression, respectively. **(B)** qPCR results. **p<0.01 vs POF+Saline; t test; n=4. **(C)** ELISA results for interleukin, TNFα, and IFNγin the peripheral blood of mice in each group. (D) Western blotting assay results. *p<0.05 vs POF+Saline; **p<0.01 vs POF+Saline; t test; n=3.

Further analysis of immune system-related data from the RNA-Seq results revealed that the mRNA expression of the interleukin (*IL1b*, *IL6*, *IL4*), NFκB (*Nfkbid*), and TNF (*Tnf*, *Tnfsf14*) families was significantly downregulated in the POF+TP-5 group compared to that in the POF+Saline group (Figure 5A, Table S1), whereas expression of the TGFβ-Smad signaling pathway family (*Smad9*) was significantly increased (Figure 5A, Table S1). Sequencing results suggested that TP-5 significantly inhibits the expression of interleukin and tumor necrosis factor genes. The results of qPCR were in good agreement with the above RNA-Seq results (Figure 5B). And, the ELISA results showed that IL-1β, IL-6, IL-22, TNFα, and IFNγ levels in the peripheral blood of the TP-5 intervention group were significantly lower than those in the POF+Saline group (Figure 5C). Meanwhile, the results of western blot showed that expression levels of NFκB inperipheral blood mononuclear cellnucleuswere significantly downregulated in the POF+TP-5 group compared to them in the POF+Saline group (Figure 5D). These results showed that TP-5 significantly improves the level of immunoregulatory factors.

### TP-5 promotes Mφ2 proportion in the ovary and corrects POF by regulating the expression of the BMP4/Smad9 signalling pathway

**Figure 6:**
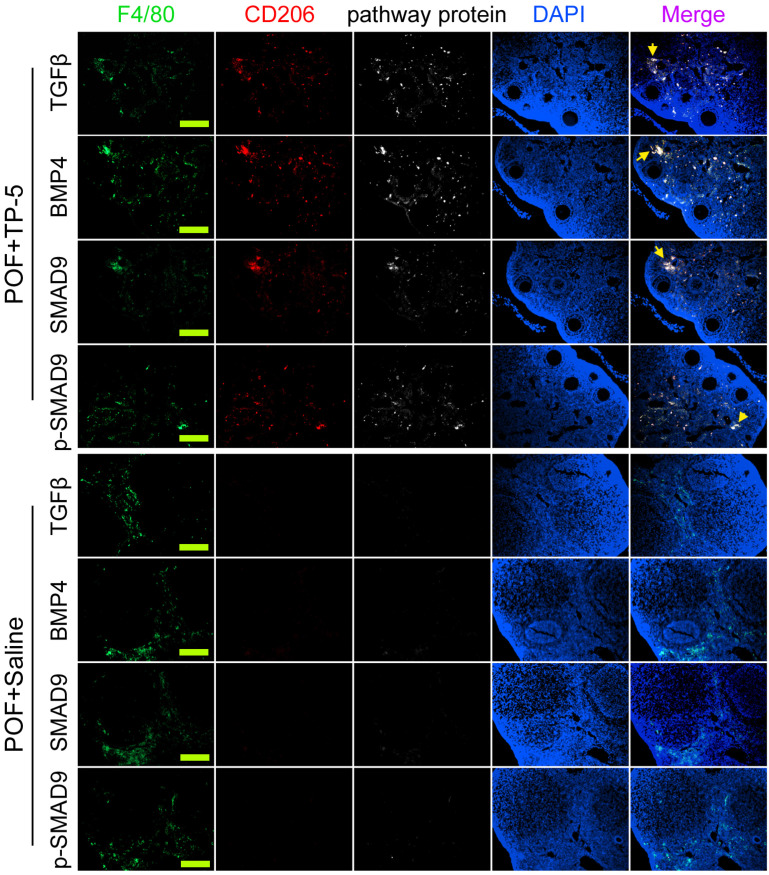
** Immunofluorescence staining of key proteins of the BMP4/Smad9 signaling pathway of Mø2 cells in ovarian tissue.** The pathway protein refers to TGFβ, BMP4, SMAD9, p-SMAD9 (phosphorylated SMAD9). Scale bars: 30 µm.

**Figure 7:**
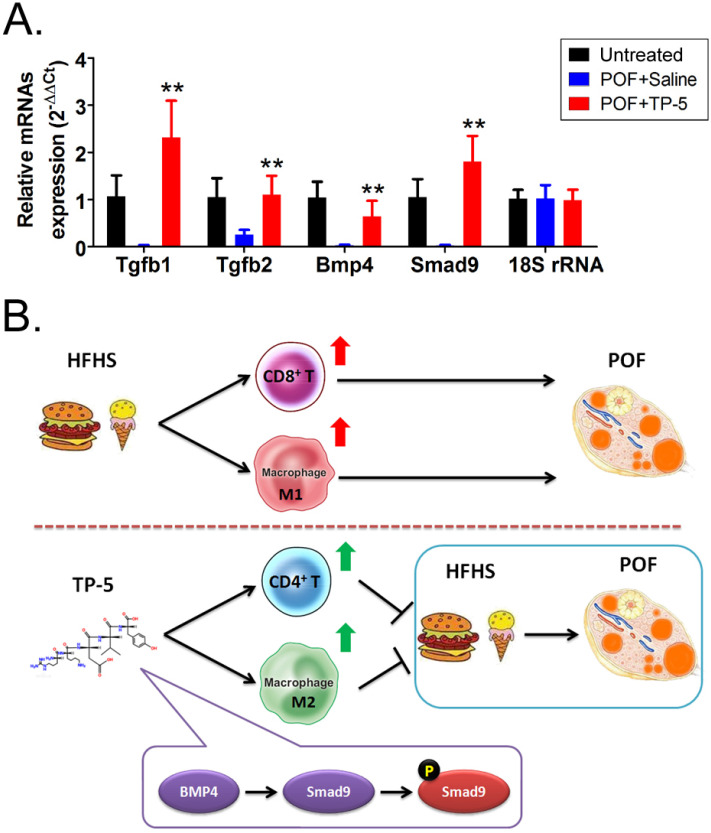
** TP-5 treatment of murine POF via activation of the BMP4/Smad9 signalling pathway. (A)** qPCR results. **p<0.01 vs POF+Saline; t test; n=4. **(B)** The mechanism underlying TP-5 treatment of murine POF via activation of the BMP4/Smad9 signalling pathway.

Immunofluorescence staining showed that the proportions of F4/80^+^/CD206^+^/BMP4^+^, F4/80^+^/CD206^+^/TGFβ^+^, F4/80^+^/CD68^+^/Smad9^+^, and F4/80^+^/CD68^+^/p-Smad9^+^ (phosphorylated Smad9) cells in the ovarian tissues of TP-5-intervention mice were significantly lower from those in the control group (Figure 6). And, qPCR results indicated that the expression levels of Tgfβ, Bmp4 and Smad9 in the POF+TP-5 group were significantly lowerthan them in the POF+Saline group (Figure 7A). These results showed that TP-5 promotes the correction of POF by activating the BMP4/Smad9 signalling pathway to promote Mφ2 polarization in the ovaries.

## Discussion

POF is both a reproductive endocrine disorder and in a broad sense, a form of pathological aging of the reproductive system 1-5. The pathogenesis of POF involves many factors, and inflammatory activity in the ovary may be one of the main causes of POF in humans 19. Therefore, targeting the regulation of immune inflammatory responses to a steady state is a rational strategy for POF treatment. On the other hands, a HFHS diet has been recently reported to lead to ovarian dysfunction, which contributes to decreased female reproductive capacity 8, 10-13, 18.several recent studies have focused on the adverse effects of a HFHS diet on health. Consumption of a HFHS diet can promote the development of various diseases, including cardiovascular disease, tumors, and diabetes via increasing the lipid oxidative stress injury 7, 9, 12. CD4+ T cells, CD8+ T cells, and macrophages play essential roles in immune responses. T lymphocytes, which are derived from bone marrow-derived lymphoid stem cells, differentiate and mature in the thymus and are distributed to the distant immune organs and tissues via the lymphatic and blood circulation systems to exert their immune functions 20, 21. T cells include subsets, such as helper T cells (CD4+ T, Th), which support humoral and cellular immunity, and cytotoxic T cells (CD8+ T, Tc), which kill target cells 20, 21. Studies have shown lymphocytic infiltration in the ovarian tissues of patients with ovarian insufficiency, suggesting a relationship between immune inflammatory activity and ovarian function 19, 22. Mø are white blood cells derived from monocytes and located in the tissues. Both Mø and monocytes are phagocytic cells involved in non-specific defense (innate immunity) and specific defense (cellular immunity) in vertebrates. The primary function of Mø is to phagocytose and digest cell debris and pathogens as immotile or motile cells, and to activate the response of lymphocytes and other immune cells to pathogens 23. Wu *et al.* indicated that although the interaction between ovarian steroid hormones and pituitary gonadotropins is a major regulator of the ovarian cycle, there is significant evidence indicating that Mø act as critical support cells for optimal fertility due to their nutritional function in reproductive tissues. The specific localization and distribution of Mø in the ovary at different stages of the menstrual cycle and their presence in human follicular fluid during ovulation suggest that macrophages play different roles in the ovarian functions, including follicular development and tissue reorganization during ovulation, and corpus luteum formation, degeneration 23-25. Thus, T cells and Mø are closely related to ovarian function.

A growing number of studies have pointed out that cytokines, especially inflammatory factors, can induce ovarian tissue or follicle aging and death 26-29. Paul F Terranova et al reported that TNFα induced serum amyloid A 3 among acute-phase proteins in mouse granulosa cells by activating NF-κBsignaling via p55 TNFα receptor type 1 28. Meanwhile, Jodi Anne Flaws also reported that TNF and both receptors (TNFRSF1A and TNFRSF1B) are expressed in neonatal mouse ovaries and that TNF promotes oocyte death in neonatal ovaries *in vitro* 29. Their results and other studies also indicated that TNFα has been shown directly to be a trigger of ovarian aging at the level of primordial follicles 28, 29. Our research found that the concentrations of some inflammatory factors (IL-1β, TNFα, etc.) in the peripheral blood mononuclear cell of POF mice group were elevated significantly. According to above reports, we have reason to believe that HFHS promoted ovarian aging through inflammatory factors, especially IL-1β, TNFα and so on. In addition, some studies have reported that a HFHS diet increases the risk of obesity, tumor formation, and cardiovascular disease 7-9 and adversely affects ovarian function and ovum quality 10-12. The HFHS diet significantly induced abnormal glucose and lipid metabolism, increased oxidative free radicals and accelerated cell aging 10-12. Meanwhile, The oxidative stress reaction derived to the HFHS diet lead to over activation of immune cells and long-term release of inflammatory factors, and eventually lead to immune imbalance 10-12. However, some studies reported that TP-5 had therapeutic effects on autoimmune diseases such as rheumatoid arthritis, type II diabetes, and female climacteric syndrome 14-18. Since the HFHS diet and TP-5 have effects by regulating immune activity, we have reason to believe that TP-5 could also treat POF derived to the HFHS diet 10-12. When mice were treated to TP-5, the phenotype of premature ovarian failure had significantly alleviated, and at the same time, the concentration of inflammatory factors in peripheral blood mononuclear cell decreased significantly. It can be seen that the concentration of inflammatory factors and the phenotype of POF were weakened by TP-5 at the same time. Therefore, we suggested that TP-5 alleviated the pathological aging of ovary by reducing the inflammatory factors.According to some reports, TP-5 effectively regulates immune cell activity and homeostasis and the release of immune factors; however, there are no reports on its efficacy in the treatment of POF, and it is unclear whether it can alleviate the symptoms of POF by targeting the regulation of T cells and Mø. One of the roles of TP-5 is to induce T cell differentiation by selectively inducing the conversion of Thy-1-prethymocytes into Thy-1+ T cells 14-18, a process mediated by elevated intracellular cAMP levels. TP-5 also enhances the phagocytic function of macrophages, enhances the enzymatic activity and phagocytosis of polymorphonuclear neutrophils, increases the number of circulating antibodies, and augments the immune function of red blood cells 14-18. In our study, we found that TP-5 significantly reduced the proportions of activated T cells (CD3+/CD8+) and Mφ1 (F4/80+/CD68+) and the expression of inflammatory factors in POF mice decreased with the number of inflammatory cells.

Finally, in light of the evidence showing that TP-5 modulated Mø proportion and activity (Mø1→Mø2), we investigated the molecular biological mechanism of this process. We found overexpression of BMP4/Smad9 signalling pathway molecules in the ovarian Mø2 cells in the TP-5 group. TGF-β superfamily signalling plays an important role in the regulation of cell growth, differentiation, and development in numerous biological systems. In general, signal transduction is initiated by ligand-induced oligomerization of the serine/threonine receptor kinases and phosphorylation of cytoplasmic signal transduction molecules, such Smad2 and Smad3 in the TGF-β/activin pathway, or Smad1/5/9 in the bone morphogenetic protein (BMP) pathway. Phosphorylation of the carboxyl-terminus of Smads by the activated receptor results in binding to the common signal transduction factor, Smad4, and translocation into the nucleus. Binding of activated Smads to transcription factors results in cell-specific transcriptional regulation of various biological effects 30-33. Therefore, we hypothesize that TP-5 converts Mø1 into Mø2 in POF ovaries by activating the BMP4/Smad9 signalling pathway in ovarian Mø cells (Figure 7B).

In summary, TP-5 attenuated activation and proportion of immune cells, and weakens the release of inflammatory factors and lipid oxidative stress injury by reducing BMP4/Smad9 signalling in the ovaries of POF mice, thereby improving ovarian tissue function.

## Supplementary Material

Supplementary figures and tables.
