# Supplementary material for: Thymopentin treatment of murine premature ovarian failure via attenuation of immune cell activity and promotion of the BMP4/Smad9 signalling pathway
Source: Int J Med Sci. 2021 Aug 21;18(15):3544–55. doi: 10.7150/ijms.61975 (PMC8436114; doi:10.7150/ijms.61975)
Supplement: Supplementary file 1 — Supplementary figures and tables. [file ijmsv18p3544s1.pdf]

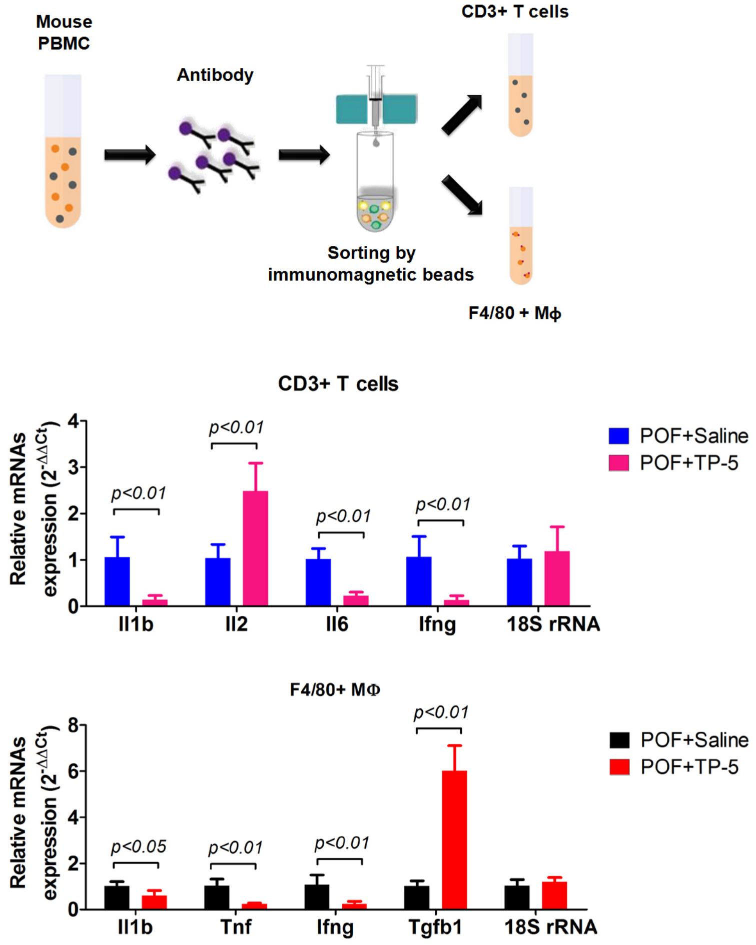
Mouse PBMC
Antibody
Sorting by immunomagnetic beads
CD3+ T cells
F4/80 + Mϕ
CD3+ T cells
Relative mRNAs expression (2-ΔΔCt)
POF+Saline
POF+TP-5
p<0.01
p<0.01
p<0.01
p<0.01
Il1b
Il2
Il6
Ifng
18S rRNA
F4/80+ MΦ
Relative mRNAs expression (2-ΔΔCt)
POF+Saline
POF+TP-5
p<0.05
p<0.01
p<0.01
p<0.01
Il1b
Tnf
Ifng
Tgfb1
18S rRNA

Table S1 Results of RNA-Seq

ID	gene_name	Annotation	Ratio (TP-5/POF)
ENSMUSG00000091736	Yy2	transcription factor YY2 [Mus musculus]	36.201
ENSMUSG00000098470	C1rb	complement C1r-B subcomponent precursor [Mus musculus]	13.503
ENSMUSG00000094475	Gm11007	KRAB box and zinc finger, C2H2 type domain containing [Mus musculus]	9.537
ENSMUSG00000024298	Zfp871	mKIAA3006 protein, partial [Mus musculus]	5.094
ENSMUSG00000078875	Gm14419	novel KRAB box and zinc finger, C2H2 type domain containing protein [Mus musculus]	4.383
ENSMUSG00000041624	Gucy1a2	guanylate cyclase soluble subunit alpha-2 [Mus musculus]	4.151
ENSMUSG00000073664	Nbeal1	neurobeachin-like protein 1 [Mus musculus]	3.906
ENSMUSG00000064357	mt-Atp6	ATP synthase F0 subunit 6 (mitochondrion) [Mus spretus]	3.824
ENSMUSG00000	Zfp169	zinc finger protein 169 isoform a [Mus	3.713

050954		musculus]	
ENSMUSG00000 009145	Dqx1	ATP-dependent RNA helicase DQX1 [Mus musculus]	3.677
ENSMUSG00000 023036	Pcdhgc4	protocadherin gamma-C4 [Mus musculus]	3.613
ENSMUSG00000 075324	Fign	fidgetin isoform 1 [Mus musculus]	3.526
ENSMUSG00000 074221	Zfp568	zinc finger protein 568 isoform 1 [Mus musculus]	3.386
ENSMUSG00000 024742	Fen1	flap structure specific endonuclease 1, isoform CRA_a, partial [Mus musculus]	3.320
ENSMUSG00000 089774	Slc5a3	sodium/myo-inositol cotransporter [Mus musculus]	3.274
ENSMUSG00000 048154	Kmt2d	histone-lysine N-methyltransferase 2D [Mus musculus]	3.242
ENSMUSG00000 024383	Map3k2	mitogen-activated protein kinase kinase kinase 2 [Mus musculus]	3.223
ENSMUSG00000 002028	Kmt2a	RecName: Full=Histone-lysine N-methyltransferase 2A; Short=Lysine N-methyltransferase 2A; AltName: Full=ALL-1; AltName: Full=Myeloid/lymphoid or mixed-lineage	3.201

		leukemia; AltName: Full=Myeloid/lymphoid or mixed-lineage leukemia protein 1; AltName: Full=Zinc finger protein HRX; Contains: RecName: Full=MLL cleavage product N320; AltName: Full=N-terminal cleavage product of 320 kDa; Short=p320; Contains: RecName: Full=MLL cleavage product C180; AltName: Full=C-terminal cleavage product of 180 kDa; Short=p180	
ENSMUSG00000051506	Wdfy4	PREDICTED: WD repeat- and FYVE domain-containing protein 4 isoform X1 [Mus musculus]	3.127
ENSMUSG00000057894	Zfp329	PREDICTED: zinc finger protein 329 isoform X1 [Mus musculus]	3.082
ENSMUSG00000067336	Bmpr2	bone morphogenetic protein receptor type-2 precursor [Mus musculus]	3.081
ENSMUSG00000037533	Rapgef6	PREDICTED: rap guanine nucleotide exchange factor 6 isoform X2 [Mus musculus]	3.071
ENSMUSG00000025094	Slc18a2	synaptic vesicular amine transporter [Mus musculus]	3.057

ENSMUSG00000057335	Cep170	PREDICTED: centrosomal protein of 170 kDa isoform X6 [Mus musculus]	3.057
ENSMUSG00000028053	Ash1l	histone-lysine N-methyltransferase ASH1L [Mus musculus]	3.054
ENSMUSG00000022419	Deptor	mKIAA4200 protein, partial [Mus musculus]	3.041
ENSMUSG00000003282	Plag1	zinc finger protein PLAG1 [Mus musculus]	3.032
ENSMUSG00000017291	Taok1	serine/threonine-protein kinase TAO1 [Mus musculus]	3.021
ENSMUSG00000021514	Zfp369	neurotrophin receptor-interacting factor 2 [Mus musculus]	3.015
ENSMUSG00000024294	Mib1	E3 ubiquitin-protein ligase MIB1 [Mus musculus]	2.954
ENSMUSG00000019841	Rev3l	DNA polymerase zeta catalytic subunit [Mus musculus]	2.932
ENSMUSG00000034647	Ankrd12	ankyrin repeat domain-containing protein 12 [Mus musculus]	2.931
ENSMUSG00000038267	Slc22a23	solute carrier family 22 member 23 [Mus musculus]	2.921
ENSMUSG00000026648	Dclre1c	protein artemis isoform 1 [Mus musculus]	2.915

ENSMUSG00000047793	Sned1	sushi, nidogen and EGF-like domain-containing protein 1 precursor [Mus musculus]	2.912
ENSMUSG00000034158	Lrrc58	leucine-rich repeat-containing protein 58 [Mus musculus]	2.877
ENSMUSG00000004328	Hif3a	hypoxia-inducible factor 3-alpha isoform 1 [Mus musculus]	2.865
ENSMUSG00000078816	Prkcg	protein kinase C gamma type isoform 1 [Mus musculus]	2.852
ENSMUSG00000061436	Hipk2	homeodomain-interacting protein kinase 2 isoform 2 [Mus musculus]	2.846
ENSMUSG00000037416	Dmxl1	dmX-like protein 1 [Mus musculus]	2.844
ENSMUSG00000038056	Kmt2c	histone-lysine N-methyltransferase 2C [Mus musculus]	2.797
ENSMUSG00000041245	Wnk3	serine/threonine-protein kinase WNK3 isoform 1 [Mus musculus]	2.790
ENSMUSG00000032253	Phip	PH-interacting protein [Mus musculus]	2.781
ENSMUSG00000017418	Arl5b	ADP-ribosylation factor-like protein 5B [Mus musculus]	2.751
ENSMUSG00000	Lnpep	leucyl-cystinyl aminopeptidase [Mus	2.738

023845		musculus]	
ENSMUSG00000058006	Mdn1	PREDICTED: midasin isoform X1 [Mus musculus]	2.738
ENSMUSG00000022629	Kif21a	kinesin-like protein KIF21A isoform 1 [Mus musculus]	2.713
ENSMUSG00000068130	Zfp442	PREDICTED: zinc finger protein 442 isoform X1 [Mus musculus]	2.691
ENSMUSG00000019726	Lyst	lysosomal-trafficking regulator [Mus musculus]	2.686
ENSMUSG00000034160	Ogt	UDP-N-acetylglucosamine--peptide N-acetylglucosaminyltransferase 110 kDa subunit isoform 1 [Mus musculus]	2.683
ENSMUSG00000078866	Gm14420	KRAB box and zinc finger C2H2 type domain containing protein-like [Mus musculus]	2.674
ENSMUSG00000041268	Dmxl2	dmX-like protein 2 [Mus musculus]	2.667
ENSMUSG00000048279	Sacs	PREDICTED: sacsin isoform X3 [Mus musculus]	2.662
ENSMUSG00000033004	Mycbp2	E3 ubiquitin-protein ligase MYCBP2 [Mus musculus]	2.654
ENSMUSG00000	Zfp708	zinc finger protein 708 isoform b [Mus	2.645

058883		musculus]	
ENSMUSG00000031292	Cdkl5	cyclin-dependent kinase-like 5 [Mus musculus]	2.634
ENSMUSG00000021287	Xrcc3	DNA repair protein XRCC3 [Mus musculus]	2.632
ENSMUSG00000031229	Atrx	transcriptional regulator ATRX [Mus musculus]	2.629
ENSMUSG00000050064	Zfp697	zinc finger protein 697 [Mus musculus]	2.623
ENSMUSG00000056608	Chd9	chromodomain-helicase-DNA-binding protein 9 isoform 1 [Mus musculus]	2.617
ENSMUSG00000063894	Zkscan8	zinc finger protein with KRAB and SCAN domains 8 isoform 1 [Mus musculus]	2.615
ENSMUSG00000063108	Zfp26	zinc finger protein 26 [Mus musculus]	2.604
ENSMUSG00000090100	Ttbk2	tau-tubulin kinase 2 isoform 1 [Mus musculus]	2.600
ENSMUSG00000050174	Nudt6	nucleoside diphosphate-linked moiety X motif 6 isoform 1 [Mus musculus]	2.594
ENSMUSG00000043940	Wdfy3	PREDICTED: WD repeat and FYVE domain-containing protein 3 isoform X2 [Mus musculus]	2.590

ENSMUSG00000026596	Abl2	Abelson tyrosine-protein kinase 2 isoform b [Mus musculus]	2.582
ENSMUSG00000037795	N4bp2	PREDICTED: NEDD4-binding protein 2 isoform X1 [Mus musculus]	2.570
ENSMUSG00000070047	Fat1	protocadherin Fat 1 precursor [Mus musculus]	2.545
ENSMUSG00000021704	Mtx3	metaxin-3 [Mus musculus]	2.533
ENSMUSG00000002265	Peg3	paternally-expressed gene 3 protein [Mus musculus]	2.525
ENSMUSG00000074867	Zfp808	zinc finger protein 80 [Mus musculus]	2.519
ENSMUSG00000051977	Prdm9	histone-lysine N-methyltransferase PRDM9 [Mus musculus]	2.517
ENSMUSG00000066687	Zbtb16	zinc finger and BTB domain-containing protein 16 [Mus musculus]	2.508
ENSMUSG00000031529	Tnks	tankyrase-1 [Mus musculus]	2.499
ENSMUSG00000002413	Braf	serine/threonine-protein kinase B-raf [Mus musculus]	2.485
ENSMUSG00000038664	Herc1	probable E3 ubiquitin-protein ligase HERC1 [Mus musculus]	2.481

ENSMUSG00000053580	Tanc2	protein TANC2 [Mus musculus]	2.479
ENSMUSG00000078651	Aoc2	retina-specific copper amine oxidase [Mus musculus]	2.446
ENSMUSG00000027009	Itga4	integrin alpha-4 precursor [Mus musculus]	2.438
ENSMUSG00000052557	Gan	gigaxonin [Mus musculus]	2.432
ENSMUSG00000032525	Nktr	PREDICTED: NK-tumor recognition protein isoform X4 [Mus musculus]	2.431
ENSMUSG00000052812	Atad2b	ATPase family AAA domain-containing protein 2B [Mus musculus]	2.430
ENSMUSG00000002107	Celf2	CUGBP Elav-like family member 2 isoform 1 [Mus musculus]	2.422
ENSMUSG00000020948	Klhl28	BTB (POZ) domain containing 5, isoform CRA_b, partial [Mus musculus]	2.401
ENSMUSG00000028370	Pappa	pregnancy-associated plasma protein-A, partial [Mus musculus]	2.400
ENSMUSG00000048732	Klhl11	kelch-like protein 11 precursor [Mus musculus]	2.393
ENSMUSG00000045962	Wnk1	serine/threonine-protein kinase WNK1 isoform 5 [Mus musculus]	2.379

ENSMUSG00000033964	Zbtb41	zinc finger and BTB domain-containing protein 41 [Mus musculus]	2.372
ENSMUSG00000034751	Mast4	microtubule-associated serine/threonine-protein kinase 4 [Mus musculus]	2.366
ENSMUSG00000018199	Trove2	60 kDa SS-A/Ro ribonucleoprotein [Mus musculus]	2.365
ENSMUSG00000073557	Ppp1r12b	protein phosphatase 1 regulatory subunit 12B [Mus musculus]	2.356
ENSMUSG00000028518	Prkaa2	5'-AMP-activated protein kinase catalytic subunit alpha-2 isoform 1 [Mus musculus]	2.353
ENSMUSG00000057230	Aak1	AP2 associated kinase 1 [Mus musculus]	2.350
ENSMUSG00000051278	Zgrf1	protein ZGRF1 [Mus musculus]	2.350
ENSMUSG00000030451	Herc2	E3 ubiquitin-protein ligase HERC2 [Mus musculus]	2.333
ENSMUSG00000073016	Uprt	uracil phosphoribosyltransferase homolog [Mus musculus]	2.332
ENSMUSG00000038384	Setd1b	PREDICTED: histone-lysine N-methyltransferase SETD1B isoform X1	2.331

		[Mus musculus]	
ENSMUSG00000026384	Ptpn4	tyrosine-protein phosphatase non-receptor type 4 [Mus musculus]	2.321
ENSMUSG00000017550	Atad5	ATPase family AAA domain-containing protein 5 [Mus musculus]	2.317
ENSMUSG00000103037	Pcdhgb1	protocadherin gamma-B1 precursor [Mus musculus]	2.312
ENSMUSG00000067928	Zfp760	zinc finger protein 760 [Mus musculus]	2.307
ENSMUSG00000022812	Gsk3b	glycogen synthase kinase-3 beta isoform 2 [Mus musculus]	2.292
ENSMUSG00000040021	Lats1	serine/threonine-protein kinase LATS1 [Mus musculus]	2.292
ENSMUSG00000062627	Mysm1	histone H2A deubiquitinase MYSM1 [Mus musculus]	2.291
ENSMUSG00000090112	Shprh	E3 ubiquitin-protein ligase SHPRH isoform a [Mus musculus]	2.262
ENSMUSG00000003226	Ranbp2	E3 SUMO-protein ligase RanBP2 [Mus musculus]	2.259
ENSMUSG00000020580	Rock2	rho-associated protein kinase 2 [Mus musculus]	2.251
ENSMUSG00000	Zfp101	zinc finger protein 101 [Mus musculus]	2.246

055240			
ENSMUSG00000 002617	Zfp40	zinc finger protein 40 [Mus musculus]	2.246
ENSMUSG00000 052331	Ankrd4 4	PREDICTED: serine/threonine-protein phosphatase 6 regulatory ankyrin repeat subunit B isoform X4 [Mus musculus]	2.242
ENSMUSG00000 026667	Uhmk1	serine/threonine-protein kinase Kist isoform 1 [Mus musculus]	2.238
ENSMUSG00000 002109	Ddb2	DNA damage-binding protein 2 [Mus musculus]	2.238
ENSMUSG00000 024290	Rock1	rho-associated protein kinase 1 [Mus musculus]	2.230
ENSMUSG00000 026409	Pfkfb2	6-phosphofructo-2-kinase/fructose-2,6-bis phosphatase 2 isoform X5 [Mus caroli]	2.229
ENSMUSG00000 026490	Cdc42b pa	Cdc42bpa protein [Mus musculus]	2.228
ENSMUSG00000 031644	Nek1	mKIAA1901 protein, partial [Mus musculus]	2.221
ENSMUSG00000 040661	Rad54l2	Rad54 like 2 (S. cerevisiae) [Mus musculus]	2.218
ENSMUSG00000 032384	Csnk1g 1	casein kinase 1, gamma 1, partial [Mus musculus]	2.209

ENSMUSG00000054737	Zfp182	zinc finger protein 182, isoform CRA_a [Mus musculus]	2.205
ENSMUSG00000022263	Trio	triple functional domain protein [Mus musculus]	2.202
ENSMUSG00000041235	Chd7	chromodomain-helicase-DNA-binding protein 7 isoform X1 [Mus pahari]	2.190
ENSMUSG00000043535	Setx	probable helicase senataxin [Mus musculus]	2.190
ENSMUSG00000052942	Glis3	zinc finger protein GLIS3 isoform 1 [Mus musculus]	2.184
ENSMUSG00000027799	Nbea	neurobeachin [Mus musculus]	2.175
ENSMUSG00000038146	Notch3	neurogenic locus notch homolog protein 3 precursor [Mus musculus]	2.175
ENSMUSG00000037826	Ppm1k	protein phosphatase 1K, mitochondrial precursor [Mus musculus]	2.171
ENSMUSG00000053877	Srcap	helicase SRCAP [Mus musculus]	2.160
ENSMUSG00000030201	Lrp6	low-density lipoprotein receptor-related protein 6 precursor [Mus musculus]	2.156
ENSMUSG00000025949	Pikfyve	1-phosphatidylinositol 3-phosphate 5-kinase isoform 1 [Mus musculus]	2.155

ENSMUSG00000063663	Brwd3	bromodomain and WD repeat-containing protein 3 [Mus musculus]	2.143
ENSMUSG00000029238	Clock	mKIAA0334 protein, partial [Mus musculus]	2.132
ENSMUSG00000007613	Tgfbr1	TGF-beta receptor type-1 isoform X1 [Mus pahari]	2.128
ENSMUSG00000022811	Zfp148	zinc finger protein 148 [Mus musculus]	2.121
ENSMUSG00000095253	Zfp799	zinc finger protein 799 isoform 1 [Mus musculus]	2.120
ENSMUSG00000021318	Gli3	transcriptional activator GLI3 [Mus musculus]	2.119
ENSMUSG00000021488	Nsd1	histone-lysine N-methyltransferase, H3 lysine-36 and H4 lysine-20 specific [Mus musculus]	2.105
ENSMUSG00000062949	Atp11c	phospholipid-transporting ATPase 11C isoform a [Mus musculus]	2.091
ENSMUSG00000042225	Ammecr1	PREDICTED: AMME syndrome candidate gene 1 protein [Fukomys damarensis]	2.091
ENSMUSG00000046743	Fat4	protocadherin Fat 4 precursor [Mus musculus]	2.089

ENSMUSG00000027737	Slc7a11	cystine/glutamate transporter [Mus musculus]	2.089
ENSMUSG00000057110	Cntrl	PREDICTED: centriolin isoform X1 [Mus musculus]	2.082
ENSMUSG00000031433	Rbm41	RNA-binding protein 41 isoform 4 [Mus musculus]	2.075
ENSMUSG00000049076	Acap2	arf-GAP with coiled-coil, ANK repeat and PH domain-containing protein 2 [Mus musculus]	2.068
ENSMUSG00000067942	Zfp160	zinc finger protein 160 [Mus musculus]	2.061
ENSMUSG00000040433	Zbtb38	zinc finger and BTB domain-containing protein 38 [Mus musculus]	2.061
ENSMUSG00000024122	Pdpk1	3-phosphoinositide-dependent protein kinase 1 isoform X1 [Mus caroli]	2.060
ENSMUSG00000022623	Shank3	Proline rich synapse associated protein 2 [Rattus norvegicus]	2.056
ENSMUSG00000039789	Zfp597	zinc finger protein 597 [Mus musculus]	2.054
ENSMUSG00000026604	Ptpn14	tyrosine-protein phosphatase non-receptor type 14 [Mus musculus]	2.054
ENSMUSG00000	Zfp937	zinc finger protein family member [Mus	2.052

060336		musculus]	
ENSMUSG00000051285	Pcmtd1	PREDICTED: protein-L-isoaspartate O-methyltransferase domain-containing protein 1 [Microtus ochrogaster]	2.051
ENSMUSG00000078606	Gm4070	interferon-induced very large GTPase 1 [Mus musculus]	2.050
ENSMUSG00000003119	Cdk12	cyclin-dependent kinase 12 isoform X1 [Mus caroli]	2.048
ENSMUSG00000021709	Erbb2ip	erbin isoform 1 [Mus musculus]	2.042
ENSMUSG00000024276	Zfp397	zinc finger protein 397 [Mus musculus]	2.040
ENSMUSG00000066324	Impad1	inositol monophosphatase 3 [Mus musculus]	2.039
ENSMUSG00000021754	Map3k1	mitogen-activated protein kinase kinase kinase 1 [Mus musculus]	2.036
ENSMUSG00000027630	Tbl1xr1	F-box-like/WD repeat-containing protein TBL1XR1 [Mus musculus]	2.033
ENSMUSG00000041459	Tardbp	TAR DNA-binding protein 43 isoform 1 [Mus musculus]	2.031
ENSMUSG00000034206	Polq	DNA polymerase theta isoform 1 [Mus musculus]	2.030

ENSMUSG00000041440	Gk5	PREDICTED: putative glycerol kinase 5 isoform X1 [Mus musculus]	2.029
ENSMUSG00000033209	Ttc28	tetratricopeptide repeat protein 28 [Mus musculus]	2.029
ENSMUSG00000001280	Sp1	transcription factor Sp1 [Mus musculus]	2.028
ENSMUSG00000051323	Pcdh19	protocadherin-19 isoform a precursor [Mus musculus]	2.020
ENSMUSG00000025323	Sp4	trans-acting transcription factor 4, partial [Mus musculus]	2.017
ENSMUSG00000044791	Setd2	histone-lysine N-methyltransferase SETD2 [Mus musculus]	2.009
ENSMUSG00000050192	Eif5a2	LOW QUALITY PROTEIN: eukaryotic translation initiation factor 5A-2 [Cavia porcellus]	2.007
ENSMUSG00000003929	Zfp81	zinc finger protein 81 [Mus musculus]	2.006
ENSMUSG00000051790	Nlgn2	neuroligin-2 precursor [Mus musculus]	2.005
ENSMUSG00000060510	Zfp266	zinc finger protein 426-like [Mus musculus]	2.004
ENSMUSG00000	Igf1r	insulin-like growth factor 1 receptor	1.998

005533		precursor [Mus musculus]	
ENSMUSG00000 021870	Slmap	sarcolemmal membrane-associated protein isoform 1 [Mus musculus]	1.994
ENSMUSG00000 024241	Sos1	son of sevenless homolog 1 [Mus musculus]	1.989
ENSMUSG00000 037111	Setd7	mKIAA1717 protein, partial [Mus musculus]	1.988
ENSMUSG00000 074220	Zfp382	zinc finger protein 382 [Mus musculus]	1.986
ENSMUSG00000 032332	Col12a1	collagen alpha-1(XII) chain isoform X3 [Mus caroli]	1.985
ENSMUSG00000 030759	Far1	fatty acyl-CoA reductase 1 isoform 1 [Mus musculus]	1.985
ENSMUSG00000 024085	Man2a1	alpha-mannosidase 2 [Mus musculus]	1.978
ENSMUSG00000 031004	Mki67	proliferation marker protein Ki-67 [Mus musculus]	1.976
ENSMUSG00000 045868	Gvin1	interferon-induced very large GTPase 1 [Mus musculus]	1.974
ENSMUSG00000 024462	Gabbr1	PREDICTED: gamma-aminobutyric acid type B receptor subunit 1 isoform X1 [Rattus norvegicus]	1.970

ENSMUSG00000014786	Slc9a5	sodium/hydrogen exchanger 5 precursor [Mus musculus]	1.969
ENSMUSG00000032097	Ddx6	DEAD (Asp-Glu-Ala-Asp) box polypeptide 6, isoform CRA_a, partial [Mus musculus]	1.965
ENSMUSG00000025092	Hspa12a	heat shock 70 kDa protein 12A isoform 2 [Mus musculus]	1.963
ENSMUSG00000062901	Klhl24	kelch-like protein 24 [Mus musculus]	1.961
ENSMUSG00000014498	Ankrd52	serine/threonine-protein phosphatase 6 regulatory ankyrin repeat subunit C [Mus musculus]	1.957
ENSMUSG00000032293	Ireb2	iron-responsive element-binding protein 2 [Mus musculus]	1.956
ENSMUSG00000005534	Insr	insulin receptor isoform A preproprotein [Mus musculus]	1.951
ENSMUSG00000054051	Ercc6	DNA excision repair protein ERCC-6 [Mus musculus]	1.946
ENSMUSG00000052920	Prkg1	cGMP-dependent protein kinase 1 beta isoform [Mus musculus]	1.944
ENSMUSG00000024077	Strn	striatin [Mus musculus]	1.941

ENSMUSG00000043019	Edem3	PREDICTED: ER degradation-enhancing alpha-mannosidase-like protein 3 isoform X2 [Mus musculus]	1.940
ENSMUSG00000038095	Sbno1	protein strawberry notch homolog 1 isoform a [Mus musculus]	1.937
ENSMUSG00000035898	Uba6	ubiquitin-like modifier-activating enzyme 6 [Mus musculus]	1.936
ENSMUSG00000026674	Ddr2	discoidin domain-containing receptor 2 precursor [Mus musculus]	1.934
ENSMUSG00000053965	Pde5a	cGMP-specific 3',5'-cyclic phosphodiesterase [Mus musculus]	1.927
ENSMUSG00000033792	Atp7a	copper-transporting ATPase 1 isoform 1 [Mus musculus]	1.923
ENSMUSG00000022360	Atad2	ATPase family AAA domain-containing protein 2 [Mus musculus]	1.921
ENSMUSG00000028381	Ugcg	ceramide glucosyltransferase [Mus musculus]	1.917
ENSMUSG00000022533	Atp13a3	probable cation-transporting ATPase 13A3 isoform 1 [Mus musculus]	1.907
ENSMUSG00000022228	Zscan26	zinc finger and SCAN domain-containing protein 26 isoform a [Mus musculus]	1.901
ENSMUSG00000	Hipk1	homeodomain-interacting protein kinase 1	1.900

008730		isoform 1 [Mus musculus]	
ENSMUSG00000 051098	Mblac2	metallo-beta-lactamase domain-containing protein 2 [Mus musculus]	1.898
ENSMUSG00000 022801	Lrch3	leucine-rich repeat and calponin homology domain-containing protein 3 isoform 4 [Mus musculus]	1.896
ENSMUSG00000 028399	Ptprd	RecName: Full=Receptor-type tyrosine-protein phosphatase delta; Short=Protein-tyrosine phosphatase delta; Short=R-PTP-delta; Flags: Precursor	1.896
ENSMUSG00000 024140	Epas1	endothelial PAS domain-containing protein 1 [Mus musculus]	1.896
ENSMUSG00000 047414	Flrt2	leucine-rich repeat transmembrane protein FLRT2 precursor [Mus musculus]	1.895
ENSMUSG00000 067780	Pi15	peptidase inhibitor 15 precursor [Mus musculus]	1.895
ENSMUSG00000 095440	Fignl2	putative fidgetin-like protein 2 [Mus musculus]	1.890
ENSMUSG00000 032913	Lrig2	leucine-rich repeats and immunoglobulin-like domains protein 2 isoform 1 precursor [Mus musculus]	1.889
ENSMUSG00000	Zfp236	PREDICTED: zinc finger protein 236	1.888

041258		isoform X1 [Mus musculus]	
ENSMUSG00000060224	Pyroxd2	pyridine nucleotide-disulfide oxidoreductase domain-containing protein 2 [Mus musculus]	1.882
ENSMUSG00000021112	Mpp5	MAGUK p55 subfamily member 5 [Mus musculus]	1.882
ENSMUSG00000035847	Ids	iduronate 2-sulfatase precursor [Mus musculus]	1.881
ENSMUSG00000078671	Chd2	chromodomain-helicase-DNA-binding protein 2 [Mus musculus]	1.870
ENSMUSG00000025892	Gria4	glutamate receptor 4 isoform X3 [Mus pahari]	1.867
ENSMUSG00000058624	Gda	guanine deaminase [Mus musculus]	1.866
ENSMUSG00000027878	Notch2	neurogenic locus notch homolog protein 2 precursor [Mus musculus]	1.866
ENSMUSG00000038205	Prkab2	5'-AMP-activated protein kinase subunit beta-2 [Mus musculus]	1.864
ENSMUSG00000039529	Atp8b1	phospholipid-transporting ATPase IC [Mus musculus]	1.862
ENSMUSG00000053626	Tll1	tolloid-like protein 1 precursor [Mus musculus]	1.857

ENSMUSG00000004317	Clcn5	H(+)/Cl(-) exchange transporter 5 isoform 2 [Mus musculus]	1.852
ENSMUSG00000037541	Shank2	PREDICTED: SH3 and multiple ankyrin repeat domains protein 2 isoform X13 [Mus musculus]	1.852
ENSMUSG00000040249	Lrp1	prolow-density lipoprotein receptor-related protein 1 precursor [Mus musculus]	1.851
ENSMUSG00000027087	Itgav	integrin alpha-V precursor [Mus musculus]	1.846
ENSMUSG00000037926	Ssh2	protein phosphatase Slingshot homolog 2 isoform 2 [Mus musculus]	1.843
ENSMUSG00000031386	Hcfc1	PREDICTED: host cell factor 1 isoform X1 [Mus musculus]	1.842
ENSMUSG00000067851	Arfgef1	brefeldin A-inhibited guanine nucleotide-exchange protein 1 [Mus musculus]	1.842
ENSMUSG00000020362	Cnot6	CCR4-NOT transcription complex subunit 6 [Rattus norvegicus]	1.842
ENSMUSG00000031284	Pak3	serine/threonine-protein kinase PAK 3 isoform C [Mus musculus]	1.841
ENSMUSG00000028763	Hspg2	basement membrane-specific heparan sulfate proteoglycan core protein precursor	1.841

		[Mus musculus]	
ENSMUSG00000096433	Gm4944	predicted gene 4944 [Mus musculus]	1.840
ENSMUSG00000019907	Ppp1r12a	protein phosphatase 1 regulatory subunit 12A [Mus musculus]	1.837
ENSMUSG00000028233	Tgs1	trimethylguanosine synthase [Mus musculus]	1.837
ENSMUSG00000035569	Ankrd11	ankyrin repeat domain-containing protein 11 [Mus musculus]	1.835
ENSMUSG00000034731	Dgkh	diacylglycerol kinase eta isoform 1 [Mus musculus]	1.834
ENSMUSG00000020721	Helz	probable helicase with zinc finger domain [Mus musculus]	1.833
ENSMUSG00000018800	Abca5	ATP-binding cassette sub-family A member 5 [Mus musculus]	1.830
ENSMUSG00000036676	Tmtc3	transmembrane and TPR repeat-containing protein 3 isoform 1 [Mus musculus]	1.827
ENSMUSG00000055884	Fancm	Fanconi anemia group M protein homolog [Mus musculus]	1.826
ENSMUSG00000021668	Polk	DNA polymerase kappa isoform a [Mus musculus]	1.825
ENSMUSG00000	Suv420	histone-lysine N-methyltransferase	1.820

045098	h1	KMT5B isoform a [Mus musculus]	
ENSMUSG00000029505	Ep400	E1A-binding protein p400 isoform 1 [Mus musculus]	1.819
ENSMUSG00000037400	Atp11b	probable phospholipid-transporting ATPase IF [Mus musculus]	1.819
ENSMUSG00000028414	Fktn	PREDICTED: fukutin isoform X1 [Mus musculus]	1.816
ENSMUSG00000055065	Ddx17	Putative ATP-dependent RNA helicase DDX17 [Fukomys damarensis]	1.814
ENSMUSG00000057133	Chd6	chromodomain-helicase-DNA-binding protein 6 [Mus musculus]	1.813
ENSMUSG00000022914	Brwd1	PREDICTED: bromodomain and WD repeat-containing protein 1 isoform X1 [Mus musculus]	1.809
ENSMUSG00000040520	Manea	glycoprotein endo-alpha-1,2-mannosidase [Mus musculus]	1.808
ENSMUSG00000070639	Lrrc8b	volume-regulated anion channel subunit LRRC8B [Mus musculus]	1.808
ENSMUSG00000079260	Tmppe	transmembrane protein with metallophosphoesterase domain [Mus musculus]	1.806
ENSMUSG00000	Acvr2a	activin receptor type-2A isoform X1 [Mus	1.804

052155		pahari]	
ENSMUSG00000031365	Zfp275	zinc finger protein 275 isoform 1 [Mus musculus]	1.804
ENSMUSG00000066800	Rnasel	2-5A-dependent ribonuclease [Mus musculus]	1.803
ENSMUSG00000039607	Rbms3	RNA-binding motif, single-stranded-interacting protein 3 isoform 4 [Mus musculus]	1.802
ENSMUSG00000055204	Ankrd17	ankyrin repeat domain-containing protein 17 isoform a [Mus musculus]	1.798
ENSMUSG00000032855	Pkd1	polycystin-1 precursor [Mus musculus]	1.798
ENSMUSG00000035247	Hectd1	RecName: Full=E3 ubiquitin-protein ligase HECTD1; AltName: Full=HECT domain-containing protein 1; AltName: Full=HECT-type E3 ubiquitin transferase HECTD1; AltName: Full=Protein open mind	1.796
ENSMUSG00000017376	Nlk	serine/threonine-protein kinase NLK [Mus musculus]	1.791
ENSMUSG00000022883	Robo1	roundabout homolog 1 precursor [Mus musculus]	1.791

ENSMUSG00000020715	Ern1	serine/threonine-protein kinase/endoribonuclease IRE1 precursor [Mus musculus]	1.786
ENSMUSG00000000823	Znf512b	Znf512b protein, partial [Mus musculus]	1.785
ENSMUSG00000023026	Dip2b	disco-interacting protein 2 homolog B isoform 1 [Mus musculus]	1.777
ENSMUSG00000069743	Zfp820	zinc finger protein 820 [Mus musculus]	1.774
ENSMUSG00000069769	Msi2	RNA-binding protein Musashi homolog 2 isoform 1 [Mus musculus]	1.770
ENSMUSG00000035878	Hykk	RIKEN cDNA C630028N24, isoform CRA_a [Mus musculus]	1.770
ENSMUSG00000033624	Pdpr	pyruvate dehydrogenase phosphatase regulatory subunit, mitochondrial precursor [Mus musculus]	1.770
ENSMUSG00000023852	Chd1	DNA-binding protein [Mus musculus]	1.769
ENSMUSG00000020883	Fbxl20	mKIAA4147 protein, partial [Mus musculus]	1.765
ENSMUSG00000014547	Wdfy2	WD repeat and FYVE domain-containing protein 2 [Mus musculus]	1.764

ENSMUSG00000028080	Lrba	lipopolysaccharide-responsive and beige-like anchor protein isoform alpha [Mus musculus]	1.763
ENSMUSG00000037921	Ddx60	probable ATP-dependent RNA helicase DDX60 [Mus musculus]	1.763
ENSMUSG00000029106	Add1	alpha-adducin isoform 1 [Mus musculus]	1.763
ENSMUSG00000028197	Col24a1	collagen alpha-1(XXIV) chain isoform 1 precursor [Mus musculus]	1.762
ENSMUSG00000041642	Kif21b	kinesin-like protein KIF21B [Mus musculus]	1.759
ENSMUSG00000028630	Dyrk2	dual specificity tyrosine-phosphorylation-regulated kinase 2 [Mus musculus]	1.759
ENSMUSG00000067931	Zfp948	zinc finger protein 948 [Mus musculus]	1.757
ENSMUSG00000030930	Chst15	mKIAA0598 protein, partial [Mus musculus]	1.754
ENSMUSG00000027130	Slc12a6	solute carrier family 12 member 6 isoform 2 [Mus musculus]	1.752
ENSMUSG00000015533	Itga2	VLA-2 homologue [Mus musculus]	1.749

ENSMUSG00000022822	Abcc5	multidrug resistance-associated protein 5 isoform 1 [Mus musculus]	1.747
ENSMUSG00000056124	B4galt6	beta-1,4-galactosyltransferase 6 [Mus musculus]	1.746
ENSMUSG00000027340	Slc23a2	solute carrier family 23 member 2 isoform a [Mus musculus]	1.739
ENSMUSG00000027580	Helz2	PREDICTED: helicase with zinc finger domain 2 isoform X3 [Mus musculus]	1.737
ENSMUSG00000039633	Lonrf1	LON peptidase N-terminal domain and RING finger protein 1 [Mus musculus]	1.734
ENSMUSG00000025314	Ptprj	receptor-type tyrosine-protein phosphatase eta isoform 1 [Mus musculus]	1.734
ENSMUSG00000015243	Abca1	ATP-binding cassette sub-family A member 1 [Mus musculus]	1.733
ENSMUSG00000036093	Arl5a	ADP-ribosylation factor-like protein 5A [Mus musculus]	1.733
ENSMUSG00000005802	Slc30a4	zinc transporter 4 isoform a [Mus musculus]	1.733
ENSMUSG00000031309	Rps6ka3	ribosomal protein S6 kinase alpha-3 isoform 1 [Mus musculus]	1.730
ENSMUSG00000033565	Rbfox2	RNA binding protein fox-1 homolog 2 isoform 1 [Mus musculus]	1.725

ENSMUSG00000028246	Faxc	Thioredoxin-like fold containing protein [Cricetulus griseus]	1.725
ENSMUSG00000021413	Prpf4b	PREDICTED: serine/threonine-protein kinase PRP4 homolog [Peromyscus maniculatus bairdii]	1.725
ENSMUSG00000031441	Atp11a	probable phospholipid-transporting ATPase IH isoform 1 [Mus musculus]	1.719
ENSMUSG00000026970	Rbms1	RNA-binding motif, single-stranded-interacting protein 1 isoform 1 [Mus musculus]	1.717
ENSMUSG00000062604	Srpk2	SRSF protein kinase 2 isoform b [Mus musculus]	1.716
ENSMUSG00000019979	Apaf1	apoptotic protease-activating factor 1 isoform 1 [Mus musculus]	1.714
ENSMUSG00000074212	Dnajb14	dnaJ homolog subfamily B member 14 [Mus musculus]	1.713
ENSMUSG00000045038	Prkce	protein kinase C epsilon type [Mus musculus]	1.713
ENSMUSG00000040565	Btaf1	TATA-binding protein-associated factor 172 [Mus musculus]	1.713
ENSMUSG00000056592	Zfp658	zinc finger protein 658 [Mus musculus]	1.712

ENSMUSG00000034724	Cnot6l	CCR4-NOT transcription complex subunit 6-like isoform 2 [Mus musculus]	1.711
ENSMUSG00000040118	Cacna2d1	voltage-dependent calcium channel subunit alpha-2/delta-1 isoform a preproprotein [Mus musculus]	1.711
ENSMUSG00000017485	Top2b	DNA topoisomerase 2-beta [Mus musculus]	1.708
ENSMUSG00000027288	Zfp106	zinc finger protein 106 [Mus musculus]	1.706
ENSMUSG00000006307	Kmt2b	PREDICTED: histone-lysine N-methyltransferase 2B isoform X1 [Mus musculus]	1.706
ENSMUSG00000016756	Cmah	cytidine monophosphate-N-acetylneuraminic acid hydroxylase isoform a [Mus musculus]	1.703
ENSMUSG00000074698	Csnk2a1	casein kinase II subunit alpha [Mus musculus]	1.700
ENSMUSG00000031608	Galnt7	N-acetylgalactosaminyltransferase 7 isoform 1 [Mus musculus]	1.700
ENSMUSG00000029992	Gfpt1	RecName: Full=Glutamine--fructose-6-phosphate aminotransferase [isomerizing] 1;	1.697

		AltName: Full=D-fructose-6-phosphate amidotransferase 1; AltName: Full=Glutamine:fructose-6-phosphate amidotransferase 1; Short=GFAT 1; Short=GFAT1; AltName: Full=Hexosephosphate aminotransferase 1	
ENSMUSG00000015944	Gatsl2	cytosolic arginine sensor for mTORC1 subunit 2 [Mus musculus]	1.695
ENSMUSG00000028369	Svep1	sushi, von Willebrand factor type A, EGF and pentraxin domain-containing protein 1 precursor [Mus musculus]	1.694
ENSMUSG00000031290	Lrch2	leucine-rich repeat and calponin homology domain-containing protein 2 isoform 1 [Mus musculus]	1.692
ENSMUSG00000054509	Parp4	poly [ADP-ribose] polymerase 4 [Mus musculus]	1.687
ENSMUSG00000028030	Tbck	TBC domain-containing protein kinase-like protein [Mus musculus]	1.684
ENSMUSG00000024597	Slc12a2	solute carrier family 12 member 2 [Mus musculus]	1.683
ENSMUSG00000027589	Pcmtd2	protein-L-isoaspartate O-methyltransferase domain-containing protein 2 isoform a	1.683

		[Mus musculus]	
ENSMUSG00000040274	Cdk6	cyclin-dependent kinase 6 [Mus musculus]	1.681
ENSMUSG00000032624	Eml4	echinoderm microtubule-associated protein-like 4 isoform 1 [Mus musculus]	1.680
ENSMUSG00000032558	Nphp3	nephrocystin-3 isoform a [Mus musculus]	1.679
ENSMUSG00000032308	Ulk3	serine/threonine-protein kinase ULK3 [Mus musculus]	1.678
ENSMUSG00000036943	Rab8b	ras-related protein Rab-8B [Rattus norvegicus]	1.677
ENSMUSG00000020687	Cdc27	PREDICTED: cell division cycle protein 27 homolog isoform X1 [Mus musculus]	1.674
ENSMUSG00000075595	Zfp652	zinc finger protein 652 [Mus musculus]	1.674
ENSMUSG00000019796	Lrp11	low-density lipoprotein receptor-related protein 11 isoform 1 precursor [Mus musculus]	1.673
ENSMUSG00000025060	Slk	STE20-like serine/threonine-protein kinase isoform 1 [Mus musculus]	1.673
ENSMUSG00000032621	Srek1	PREDICTED: splicing regulatory glutamine/lysine-rich protein 1 isoform X3	1.672

		[Peromyscus maniculatus bairdii]	
ENSMUSG00000005198	Polr2a	RNA polymerase II largest subunit [Mus musculus]	1.671
ENSMUSG00000022292	Rrm2b	ribonucleoside-diphosphate reductase subunit M2 B isoform 1 [Mus musculus]	1.671
ENSMUSG00000025810	Nrp1	neuropilin-1 isoform X1 [Mus pahari]	1.668
ENSMUSG00000027778	Ift80	intraflagellar transport protein 80 homolog [Mus musculus]	1.668
ENSMUSG00000042410	Agps	alkyldihydroxyacetonephosphate synthase, peroxisomal [Mus musculus]	1.666
ENSMUSG00000053774	Ubxn7	UBX domain-containing protein 7 [Mus musculus]	1.665
ENSMUSG00000014932	Yes1	tyrosine-protein kinase Yes [Mus musculus]	1.663
ENSMUSG00000033902	Mapkbp1	mitogen-activated protein kinase-binding protein 1 isoform 2 [Mus musculus]	1.663
ENSMUSG00000035798	Zdhhc17	palmitoyltransferase ZDHHC17 isoform 1 [Mus musculus]	1.661
ENSMUSG00000029920	Smarcad1	SWI/SNF-related matrix-associated actin-dependent regulator of chromatin subfamily A containing DEAD/H box 1	1.659

		isoform 1 [Mus musculus]	
ENSMUSG00000029516	Cit	PREDICTED: citron Rho-interacting kinase isoform X8 [Mus musculus]	1.658
ENSMUSG00000027966	Col11a1	collagen alpha-1(XI) chain preproprotein [Mus musculus]	1.657
ENSMUSG00000032243	Itga11	integrin alpha-11 precursor [Mus musculus]	1.656
ENSMUSG00000022048	Dpysl2	dihydropyrimidinase-related protein 2 [Mus musculus]	1.655
ENSMUSG00000054008	Ndst1	bifunctional heparan sulfate N-deacetylase/N-sulfotransferase 1-like protein [Cricetulus griseus]	1.654
ENSMUSG00000014496	Ankrd28	ankyrin repeat domain 28, isoform CRA_a, partial [Mus musculus]	1.651
ENSMUSG00000037369	Kdm6a	lysine-specific demethylase 6A isoform 1 [Mus musculus]	1.650
ENSMUSG00000034663	Bmp2k	BMP-2-inducible protein kinase [Mus musculus]	1.650
ENSMUSG00000028312	Smc2	structural maintenance of chromosomes protein 2 [Mus musculus]	1.648
ENSMUSG00000038774	Ascc3	activating signal cointegrator 1 complex subunit 3 [Mus musculus]	1.647

ENSMUSG00000057982	Zfp809	zinc finger protein 809 isoform 1 [Mus musculus]	1.646
ENSMUSG00000029406	Pitpnm2	membrane-associated phosphatidylinositol transfer protein 2 isoform 2 [Mus musculus]	1.646
ENSMUSG00000028086	Fbxw7	F-box and WD-40 domain protein 7, archipelago homolog (Drosophila), isoform CRA_b, partial [Mus musculus]	1.646
ENSMUSG00000020899	Pfas	phosphoribosylformylglycinamidine synthase [Mus musculus]	1.646
ENSMUSG00000037343	Taf2	transcription initiation factor TFIID subunit 2 [Mus musculus]	1.644
ENSMUSG00000021959	Lats2	large tumor suppressor 2, isoform CRA_b, partial [Mus musculus]	1.642
ENSMUSG00000031166	Wdr13	WD repeat-containing protein 13 isoform 1 [Mus musculus]	1.642
ENSMUSG00000021614	Vcan	mCG116562, isoform CRA_b, partial [Mus musculus]	1.642
ENSMUSG00000034320	Slc26a2	sulfate transporter [Mus musculus]	1.641
ENSMUSG00000029674	Limk1	LIM domain kinase 1 isoform 1 [Mus musculus]	1.641

ENSMUSG00000057551	Zfp317	zinc finger protein 317 [Mus musculus]	1.640
ENSMUSG00000046351	Zfp322a	zinc finger protein 322 [Mus musculus]	1.639
ENSMUSG00000032560	Dnajc13	dnaJ homolog subfamily C member 13 isoform 1 [Mus musculus]	1.638
ENSMUSG00000030084	Plxna1	plexin-A1 precursor [Mus musculus]	1.635
ENSMUSG00000075040	Zfp408	zinc finger protein 408 isoform 1 [Mus musculus]	1.633
ENSMUSG00000024811	Tnks2	tankyrase-2 [Mus musculus]	1.629
ENSMUSG00000029729	Zkscan1	zinc finger protein with KRAB and SCAN domains 1 isoform 1 [Mus musculus]	1.629
ENSMUSG00000052539	Magi3	membrane-associated guanylate kinase, WW and PDZ domain-containing protein 3 isoform 1 [Mus musculus]	1.629
ENSMUSG00000034154	Ino80	DNA helicase INO80 [Mus musculus]	1.629
ENSMUSG00000004085	Zak	mitogen-activated protein kinase kinase kinase 20 isoform 1 [Mus musculus]	1.628
ENSMUSG00000	Clcn3	H(+)/Cl(-) exchange transporter 3 isoform	1.628

004319		e [Mus musculus]	
ENSMUSG00000 035725	Prkx	cAMP-dependent protein kinase catalytic subunit PRKX [Mus musculus]	1.627
ENSMUSG00000 033319	Fem1c	protein fem-1 homolog C [Mus musculus]	1.626
ENSMUSG00000 036273	Lrrk2	leucine-rich repeat serine/threonine-protein kinase 2 [Mus musculus]	1.626
ENSMUSG00000 032846	Zswim6	zinc finger SWIM domain-containing protein 6 [Mus musculus]	1.625
ENSMUSG00000 024087	Cyp1b1	cytochrome P450 1B1 [Mus musculus]	1.624
ENSMUSG00000 020954	Strn3	striatin-3 isoform 1 [Mus musculus]	1.621
ENSMUSG00000 038970	Lmtk2	serine/threonine-protein kinase LMTK2 precursor [Mus musculus]	1.619
ENSMUSG00000 027797	Dclk1	serine/threonine-protein kinase DCLK1 isoform 1 [Mus musculus]	1.618
ENSMUSG00000 024614	Tmx3	protein disulfide-isomerase TMX3 precursor [Mus musculus]	1.617
ENSMUSG00000 030096	Slc6a6	sodium- and chloride-dependent taurine transporter [Mus musculus]	1.617

ENSMUSG00000056383	AI987944	mCG55139, isoform CRA_c, partial [Mus musculus]	1.617
ENSMUSG00000041915	Ammecr1l	AMMECR1-like protein isoform 2 [Mus musculus]	1.615
ENSMUSG00000044807	Zfp354c	mKIAA4218 protein, partial [Mus musculus]	1.615
ENSMUSG00000029016	Clcn6	chloride transport protein 6 [Mus musculus]	1.615
ENSMUSG00000070923	Klhl9	mKIAA1354 protein, partial [Mus musculus]	1.614
ENSMUSG00000037643	Prkci	protein kinase C iota type [Mus musculus]	1.614
ENSMUSG00000027109	Sp3	transcription factor Sp3 isoform 1 [Mus musculus]	1.613
ENSMUSG00000052040	Klf13	Krueppel-like factor 13 [Mus musculus]	1.612
ENSMUSG00000039202	Abhd2	monoacylglycerol lipase ABHD2 [Mus musculus]	1.611
ENSMUSG00000034653	Ythdc2	probable ATP-dependent RNA helicase YTHDC2 [Mus musculus]	1.609
ENSMUSG00000020273	Papolg	poly(A) polymerase gamma [Mus musculus]	1.608

ENSMUSG00000005371	Fbxo11	F-box only protein 11 isoform X2 [Mus pahari]	1.604
ENSMUSG00000072623	Zfp9	zinc finger protein 25 [Mus musculus]	1.604
ENSMUSG00000031278	Acsl4	acyl-CoA synthetase long-chain family member 4, isoform CRA_a, partial [Mus musculus]	1.603
ENSMUSG00000048410	Zfp407	zinc finger protein 407 [Mus musculus]	1.599
ENSMUSG00000021796	Bmpr1a	bone morphogenetic protein receptor type-1A precursor [Mus musculus]	1.596
ENSMUSG00000027312	Atrn	attractin preproprotein [Mus musculus]	1.592
ENSMUSG00000020661	Dnmt3a	DNA (cytosine-5)-methyltransferase 3A isoform 1 [Mus musculus]	1.589
ENSMUSG00000043090	Zfp866	zinc finger protein 866 [Mus musculus]	1.588
ENSMUSG00000019699	Akt3	RAC-gamma serine/threonine-protein kinase [Mus musculus]	1.584
ENSMUSG00000041415	Dicer1	Dicer1, Dcr-1 homolog (Drosophila), isoform CRA_b, partial [Mus musculus]	1.583
ENSMUSG00000	Tanc1	protein TANC1 isoform a [Mus musculus]	1.574

035168			
ENSMUSG00000021009	Ptpn21	tyrosine-protein phosphatase non-receptor type 21 [Mus musculus]	1.572
ENSMUSG00000026980	Ly75	lymphocyte antigen 75 precursor [Mus musculus]	1.568
ENSMUSG00000000600	Krit1	krev interaction trapped protein 1 isoform 1 [Mus musculus]	1.566
ENSMUSG00000033863	Klf9	Krueppel-like factor 9 [Mus musculus]	1.565
ENSMUSG00000022897	Dyrk1a	dual specificity tyrosine-phosphorylation-regulated kinase 1A isoform 1 [Mus musculus]	1.563
ENSMUSG00000036333	Kidins220	kinase D-interacting substrate of 220 kDa [Mus musculus]	1.561
ENSMUSG00000040351	Ankib1	mKIAA1386 protein, partial [Mus musculus]	1.560
ENSMUSG00000036959	Bcorl1	BCL-6 corepressor-like protein 1 [Mus musculus]	1.559
ENSMUSG00000024943	Smc5	mCG5312, isoform CRA_b, partial [Mus musculus]	1.557
ENSMUSG00000005397	Nid1	nidogen-1 precursor [Mus musculus]	1.555

ENSMUSG00000032252	Glce	D-glucuronyl C5-epimerase [Mus musculus]	1.555
ENSMUSG00000022601	Zbtb11	zinc finger and BTB domain-containing protein 11 [Mus musculus]	1.554
ENSMUSG00000026313	Hdac4	histone deacetylase 4 [Mus musculus]	1.553
ENSMUSG00000041313	Slc7a1	solute carrier family 7 (cationic amino acid transporter, y+ system), member 1, partial [Mus musculus]	1.552
ENSMUSG00000021111	Papola	poly(A) polymerase alpha isoform 1 [Mus musculus]	1.549
ENSMUSG00000020841	Cpd	carboxypeptidase D precursor [Mus musculus]	1.548
ENSMUSG00000070867	Trabd2b	metalloprotease TIKI2 precursor [Mus musculus]	1.548
ENSMUSG00000026781	Acbd5	acyl-CoA-binding domain-containing protein 5 isoform a precursor [Mus musculus]	1.548
ENSMUSG00000025266	Gnl3l	guanine nucleotide-binding protein-like 3-like protein [Mus musculus]	1.542
ENSMUSG00000036282	Naa30	N-alpha-acetyltransferase 30 [Mus musculus]	1.542

ENSMUSG00000022770	Dlg1	disks large homolog 1 isoform 1 [Mus musculus]	1.540
ENSMUSG00000021327	Zkscan3	zinc finger protein with KRAB and SCAN domains 3 [Mus musculus]	1.538
ENSMUSG00000053754	Chd8	chromodomain-helicase-DNA-binding protein 8 [Mus musculus]	1.536
ENSMUSG00000022797	Tfrc	transferrin receptor protein 1 [Mus musculus]	1.535
ENSMUSG00000028207	Asph	aspartyl/asparaginyl beta-hydroxylase isoform 1 [Mus musculus]	1.534
ENSMUSG00000026102	Inpp1	inositol polyphosphate 1-phosphatase [Mus musculus]	1.534
ENSMUSG00000038733	Wdr26	WD repeat-containing protein 26 [Mus musculus]	1.534
ENSMUSG00000032194	Kank2	KN motif and ankyrin repeat domain-containing protein 2 [Mus musculus]	1.533
ENSMUSG00000032601	Prkar2a	cAMP-dependent protein kinase type II-alpha regulatory subunit [Mus musculus]	1.532
ENSMUSG00000022994	Adcy6	adenylate cyclase type 6 [Mus musculus]	1.529

ENSMUSG00000013236	Ptprs	receptor-type tyrosine-protein phosphatase S isoform 1 precursor [Mus musculus]	1.529
ENSMUSG00000021147	Wdr37	WD repeat-containing protein 37 isoform a [Mus musculus]	1.528
ENSMUSG00000029231	Pdgfra	platelet-derived growth factor receptor alpha isoform 1 precursor [Mus musculus]	1.527
ENSMUSG00000044864	Ankrd50	PREDICTED: ankyrin repeat domain-containing protein 50 isoform X1 [Mus musculus]	1.525
ENSMUSG00000034422	Parp14	poly [ADP-ribose] polymerase 14 [Mus musculus]	1.525
ENSMUSG00000021745	Ptprg	receptor-type tyrosine-protein phosphatase gamma isoform 1 precursor [Mus musculus]	1.523
ENSMUSG00000020256	Aldh1l2	mitochondrial 10-formyltetrahydrofolate dehydrogenase [Mus musculus]	1.523
ENSMUSG00000032244	Fem1b	protein fem-1 homolog B [Mus musculus]	1.522
ENSMUSG00000002428	Hltf	helicase-like transcription factor [Mus musculus]	1.521
ENSMUSG00000029648	Flt1	vascular endothelial growth factor receptor 1 precursor [Mus musculus]	1.519

ENSMUSG00000037112	Sik2	serine/threonine-protein kinase SIK2 [Mus musculus]	1.518
ENSMUSG00000020385	Clk4	dual specificity protein kinase CLK4 isoform 1 [Mus musculus]	1.517
ENSMUSG00000017677	Wsb1	WD repeat and SOCS box-containing protein 1 isoform 1 [Mus musculus]	1.517
ENSMUSG00000006281	Tep1	telomerase protein component 1 [Mus musculus]	1.516
ENSMUSG00000034312	Iqsec1	PREDICTED: IQ motif and SEC7 domain-containing protein 1 isoform X7 [Mus musculus]	1.516
ENSMUSG00000005506	Celf1	CUGBP Elav-like family member 1 isoform 1 [Mus musculus]	1.510
ENSMUSG00000032393	Dpp8	dipeptidylpeptidase 8, isoform CRA_a, partial [Mus musculus]	1.510
ENSMUSG00000035469	Rcbtb1	RCC1 and BTB domain-containing protein 1 isoform 1 [Mus musculus]	1.509
ENSMUSG00000024242	Map4k3	mitogen-activated protein kinase kinase kinase kinase 3 isoform 1 [Mus musculus]	1.508
ENSMUSG00000024754	Tmem2	cell surface hyaluronidase [Mus musculus]	1.508
ENSMUSG00000	Wdr7	WD repeat-containing protein 7 isoform 1	1.507

040560		[Mus musculus]	
ENSMUSG00000018474	Chd3	chromodomain-helicase-DNA-binding protein 3 [Mus musculus]	1.506
ENSMUSG00000041734	Kirrel	kin of IRRE-like protein 1 precursor [Mus musculus]	1.506
ENSMUSG00000030265	Kras	PREDICTED: GTPase KRas isoform X1 [Mus musculus]	1.503
ENSMUSG00000025437	Usp33	ubiquitin carboxyl-terminal hydrolase 33 isoform 1 [Mus musculus]	1.502
ENSMUSG00000027993	Trim2	tripartite motif-containing protein 2 isoform 1 [Mus musculus]	1.502
ENSMUSG00000031583	Wrn	Werner syndrome ATP-dependent helicase homolog [Mus musculus]	1.500
ENSMUSG00000072825	Cep170b	centrosomal protein of 170 kDa protein B [Mus musculus]	1.499
ENSMUSG00000004591	Pkn2	serine/threonine-protein kinase N2 [Mus musculus]	1.499
ENSMUSG00000031558	Slit2	slit homolog 2 protein isoform a precursor [Mus musculus]	1.495
ENSMUSG00000015522	Arnt	aryl hydrocarbon receptor nuclear translocator isoform a [Mus musculus]	1.494
ENSMUSG00000	Cmip	C-Maf-inducing protein isoform 1 [Mus	1.494

034390		musculus]	
ENSMUSG00000020015	Cdk17	cyclin-dependent kinase 17 [Mus musculus]	1.491
ENSMUSG00000046873	Mbtps2	membrane-bound transcription factor site-2 protease [Mus musculus]	1.487
ENSMUSG00000021712	Trim23	RecName: Full=E3 ubiquitin-protein ligase TRIM23; AltName: Full=ADP-ribosylation factor domain-containing protein 1; AltName: Full=GTP-binding protein ARD-1; AltName: Full=RING-type E3 ubiquitin transferase TRIM23; AltName: Full=Tripartite motif-containing protein 23	1.486
ENSMUSG00000063362	Alg11	GDP-Man:Man(3)GlcNAc(2)-PP-Dol alpha-1,2-mannosyltransferase isoform 1 [Mus musculus]	1.485
ENSMUSG00000021470	Ercc6l2	DNA excision repair protein ERCC-6-like 2 isoform 1 [Mus musculus]	1.484
ENSMUSG00000037885	Stk35	serine/threonine kinase 35 [Mus musculus]	1.483
ENSMUSG00000026393	Nek7	serine/threonine-protein kinase Nek7 isoform 1 [Mus musculus]	1.483

ENSMUSG00000041297	Cdk13	cyclin-dependent kinase 13 isoform 1 [Mus musculus]	1.480
ENSMUSG00000051615	Rap2a	ras-related protein Rap-2a precursor [Mus musculus]	1.479
ENSMUSG00000025986	Slc39a10	PREDICTED: zinc transporter ZIP10 isoform X1 [Mus musculus]	1.478
ENSMUSG00000058793	Cds2	phosphatidate cytidylyltransferase 2 isoform a [Mus musculus]	1.477
ENSMUSG00000020790	Ankfy1	rabankyrin-5 [Mus musculus]	1.477
ENSMUSG00000024130	Abca3	ATP-binding cassette sub-family A member 3 [Mus musculus]	1.476
ENSMUSG00000029009	Mthfr	methylenetetrahydrofolate reductase isoform a [Mus musculus]	1.476
ENSMUSG00000026516	Nvl	nuclear valosin-containing protein-like [Mus musculus]	1.473
ENSMUSG00000013663	Pten	phosphatidylinositol 3,4,5-trisphosphate 3-phosphatase and dual-specificity protein phosphatase PTEN, partial [Mesocricetus auratus]	1.471
ENSMUSG00000043262	Uevld	ubiquitin-conjugating enzyme E2 variant 3 [Mus musculus]	1.470

ENSMUSG00000000628	Hk2	hexokinase-2 [Mus musculus]	1.468
ENSMUSG00000033991	Ttc37	tetratricopeptide repeat protein 37 [Mus musculus]	1.468
ENSMUSG00000022401	Xpnpep3	probable Xaa-Pro aminopeptidase 3 isoform 2 [Mus musculus]	1.468
ENSMUSG00000022911	Arl13b	ADP-ribosylation factor-like protein 13B [Mus musculus]	1.463
ENSMUSG00000026923	Notch1	neurogenic locus notch homolog protein 1 precursor [Mus musculus]	1.463
ENSMUSG00000054580	Pla2r1	phospholipase A2 receptor 1 [Mus musculus]	1.459
ENSMUSG00000040102	Klhl42	kelch-like protein 42 [Mus musculus]	1.459
ENSMUSG00000026705	Klhl20	kelch-like protein 20 [Mus musculus]	1.459
ENSMUSG00000051853	Arf3	ADP-ribosylation factor 3-like protein [Cricetulus griseus]	1.458
ENSMUSG00000019943	Atp2b1	plasma membrane calcium-transporting ATPase 1 isoform 2 [Mus musculus]	1.456
ENSMUSG00000024976	Shoc2	leucine-rich repeat protein SHOC-2 [Mus musculus]	1.455

ENSMUSG00000024079	Eif2ak2	interferon-induced, double-stranded RNA-activated protein kinase [Mus musculus]	1.453
ENSMUSG00000032826	Ank2	PREDICTED: ankyrin-2 isoform X27 [Mus musculus]	1.452
ENSMUSG00000060671	Atp8b2	phospholipid-transporting ATPase ID [Mus musculus]	1.452
ENSMUSG00000041773	Enc1	ectoderm-neural cortex protein 1 [Mus musculus]	1.452
ENSMUSG00000025584	Pde8a	high affinity cAMP-specific and IBMX-insensitive 3',5'-cyclic phosphodiesterase 8A [Mus musculus]	1.450
ENSMUSG00000054823	Whsc11 1	histone-lysine N-methyltransferase NSD3 isoform 2 [Mus musculus]	1.450
ENSMUSG00000025246	Tbl1x	PREDICTED: F-box-like/WD repeat-containing protein TBL1X isoform X1 [Peromyscus maniculatus bairdii]	1.449
ENSMUSG00000021109	Hif1a	hypoxia-inducible factor 1-alpha isoform 2 [Mus musculus]	1.448
ENSMUSG00000018334	Ksr1	kinase suppressor of Ras 1 isoform 1 [Mus musculus]	1.447
ENSMUSG00000	Kbtbd2	kelch repeat and BTB domain-containing	1.447

059486		protein 2 [Mus musculus]	
ENSMUSG00000031508	Ankrd10	ankyrin repeat domain-containing protein 10 isoform 1 [Mus musculus]	1.446
ENSMUSG00000042046	Dstyk	dual serine/threonine and tyrosine protein kinase [Mus musculus]	1.445
ENSMUSG00000034573	Ptpn13	tyrosine-protein phosphatase non-receptor type 13 [Mus musculus]	1.445
ENSMUSG00000022781	Pak2	serine/threonine-protein kinase PAK 2 [Ictidomys tridecemlineatus]	1.445
ENSMUSG00000031833	Mast3	microtubule-associated serine/threonine-protein kinase 3 [Mus musculus]	1.441
ENSMUSG00000075703	Ept1	ethanolaminephosphotransferase 1 [Mus musculus]	1.440
ENSMUSG00000021559	Dapk1	death-associated protein kinase 1 isoform 1 [Mus musculus]	1.439
ENSMUSG00000029334	Prkg2	cGMP-dependent protein kinase 2 [Mus musculus]	1.438
ENSMUSG00000005102	Eif2ak4	eIF-2-alpha kinase GCN2 isoform 1 [Mus musculus]	1.436
ENSMUSG00000061130	Ppm1b	mCG15599, isoform CRA_e, partial [Mus musculus]	1.435

ENSMUSG00000001986	Gria3	PREDICTED: glutamate receptor 3 isoform X1 [Nannospalax galili]	1.435
ENSMUSG00000005034	Prkacb	cAMP-dependent protein kinase catalytic subunit beta isoform 4 [Mus musculus]	1.432
ENSMUSG00000036894	Rap2b	ras-related protein Rap-2b precursor [Mus musculus]	1.430
ENSMUSG00000047409	Ctdspl	CTD small phosphatase-like protein [Mus musculus]	1.430
ENSMUSG00000063273	Naa15	N-alpha-acetyltransferase 15, NatA auxiliary subunit [Mus musculus]	1.424
ENSMUSG00000028413	B4galt1	beta-1,4-galactosyltransferase 1 [Mus musculus]	1.424
ENSMUSG00000028161	Ppp3ca	serine/threonine-protein phosphatase 2B catalytic subunit alpha isoform [Rattus norvegicus]	1.424
ENSMUSG00000074582	Arfgef2	brefeldin A-inhibited guanine nucleotide-exchange protein 2 [Mus musculus]	1.423
ENSMUSG00000036893	Ehmt1	histone-lysine N-methyltransferase EHMT1 isoform 1 [Mus musculus]	1.421
ENSMUSG00000050029	Rap2c	ras-related protein Rap-2c precursor [Mus musculus]	1.421

ENSMUSG00000024143	Rhoq	rho-related GTP-binding protein RhoQ precursor [Rattus norvegicus]	1.419
ENSMUSG00000053436	Mapk14	mitogen-activated protein kinase 14 isoform 2 [Mus musculus]	1.418
ENSMUSG00000024974	Smc3	structural maintenance of chromosomes protein 3 [Mus musculus]	1.417
ENSMUSG00000024789	Jak2	tyrosine-protein kinase JAK2 [Mus musculus]	1.416
ENSMUSG00000031659	Adcy7	adenylate cyclase type 7 [Mus musculus]	1.414
ENSMUSG00000054027	Nt5dc3	5'-nucleotidase domain-containing protein 3 [Mus musculus]	1.414
ENSMUSG00000044340	Phlpp1	PH domain leucine-rich repeat-containing protein phosphatase 1 [Mus musculus]	1.409
ENSMUSG00000024483	Ankhd1	ankyrin repeat and KH domain-containing protein 1 [Mus musculus]	1.408
ENSMUSG00000020152	Actr2	actin-related protein 2 [Rattus norvegicus]	1.407
ENSMUSG00000018481	Appbp2	amyloid protein-binding protein 2 [Mus musculus]	1.403
ENSMUSG00000038886	Man2a2	alpha-mannosidase 2x [Mus musculus]	1.403

ENSMUSG00000020608	Smc6	structural maintenance of chromosomes protein 6 isoform 1 [Mus musculus]	1.402
ENSMUSG00000038766	Gabpb2	GA-binding protein subunit beta-2 [Mus musculus]	1.398
ENSMUSG00000042688	Mapk6	mitogen-activated protein kinase 6 [Mus musculus]	1.396
ENSMUSG00000036450	Hif1an	hypoxia-inducible factor 1-alpha inhibitor [Mus musculus]	1.394
ENSMUSG00000025220	Mgea5	protein O-GlcNAcase [Mus musculus]	1.393
ENSMUSG00000079614	Seh1l	centrosomal protein of 192 kDa isoform X3 [Mus caroli]	1.393
ENSMUSG00000025757	Hspa4l	heat shock protein 4 like, isoform CRA_c, partial [Mus musculus]	1.391
ENSMUSG00000031561	Tenm3	teneurin-3 isoform 1 [Mus musculus]	1.390
ENSMUSG00000000787	Ddx3x	Ddx3x protein [Mus musculus]	1.388
ENSMUSG00000024978	Gpam	glycerol-3-phosphate acyltransferase 1, mitochondrial [Mus musculus]	1.387
ENSMUSG00000032570	Atp2c1	calcium-transporting ATPase type 2C member 1 isoform 1 [Mus musculus]	1.387

ENSMUSG00000046324	Ermp1	endoplasmic reticulum metallopeptidase 1 [Mus musculus]	1.383
ENSMUSG00000001773	Folh1	glutamate carboxypeptidase 2 isoform 1 [Mus musculus]	1.381
ENSMUSG00000033295	Ptprf	receptor-type tyrosine-protein phosphatase F precursor [Mus musculus]	1.379
ENSMUSG00000024006	Stk38	serine/threonine kinase 38, isoform CRA_a, partial [Mus musculus]	1.378
ENSMUSG00000027893	Ahcyl1	S-adenosylhomocysteine hydrolase-like protein 1 [Meriones unguiculatus]	1.378
ENSMUSG00000070780	Rbm47	RNA-binding protein 47 isoform a [Mus musculus]	1.376
ENSMUSG00000053819	Camk2d	PREDICTED: calcium/calmodulin-dependent protein kinase type II subunit delta isoform X1 [Jaculus jaculus]	1.374
ENSMUSG00000030509	Asb7	ankyrin repeat and SOCS box protein 7 [Mus musculus]	1.374
ENSMUSG00000024501	Dpysl3	dihydropyrimidinase-related protein 3 CRMP4b [Mus musculus]	1.373
ENSMUSG00000028676	Srsf10	neural specific sr protein NSSR 1 [Mus musculus]	1.372

ENSMUSG00000035941	Ibtk	inhibitor of Bruton tyrosine kinase isoform 1 [Mus musculus]	1.371
ENSMUSG00000015133	Lrrk1	leucine-rich repeat serine/threonine-protein kinase 1 [Mus musculus]	1.371
ENSMUSG00000029840	Mtpn	myotrophin [Mus musculus]	1.370
ENSMUSG00000030704	Rab6a	ras-related protein Rab-6A isoform 2 [Mus musculus]	1.368
ENSMUSG00000032440	Tgfbr2	TGF-beta receptor type-2 isoform 1 precursor [Mus musculus]	1.368
ENSMUSG00000050697	Prkaa1	5'-AMP-activated protein kinase catalytic subunit alpha-1 isoform 1 [Mus musculus]	1.367
ENSMUSG00000039159	Ube2h	ubiquitin-conjugating enzyme E2 H [Mus caroli]	1.366
ENSMUSG00000027770	Dhx36	ATP-dependent RNA helicase DHX36 [Mus musculus]	1.366
ENSMUSG00000037071	Scd1	acyl-CoA desaturase 1 [Mus musculus]	1.365
ENSMUSG00000020032	Nuak1	NUAK family SNF1-like kinase 1 [Mus musculus]	1.364

ENSMUSG00000 034761	Map4k5	mitogen-activated protein kinase kinase kinase kinase 5 [Mus musculus]	1.364
ENSMUSG00000 017929	B4galt5	beta-1,4-galactosyltransferase V [Mus musculus]	1.358
ENSMUSG00000 045039	Megf8	multiple epidermal growth factor-like domains protein 8 precursor [Mus musculus]	1.357
ENSMUSG00000 024921	Smarca 2	PREDICTED: probable global transcription activator SNF2L2 isoform X1 [Mus musculus]	1.353
ENSMUSG00000 042793	Lgr6	leucine-rich repeat-containing G-protein coupled receptor 6 precursor [Mus musculus]	1.337
ENSMUSG00000 021279	Cdc42b pb	serine/threonine-protein kinase MRCK beta [Mus musculus]	1.330
ENSMUSG00000 022194	Pabpn1	Polyadenylate-binding protein 2 [Cricetulus griseus]	1.328
ENSMUSG00000 020532	Acaca	PREDICTED: acetyl-CoA carboxylase 1 isoform X2 [Mus musculus]	1.321
ENSMUSG00000 020122	Egfr	epidermal growth factor receptor isoform 1 precursor [Mus musculus]	1.279
ENSMUSG00000	Csnk1a	casein kinase I isoform X6	1.277

024576	1	[Heterocephalus glaber]	
ENSMUSG00000 022604	Cep97	centrosomal protein of 97 kDa isoform 1 [Mus musculus]	1.270
ENSMUSG00000 019790	Stxbp5	PREDICTED: syntaxin-binding protein 5 isoform X1 [Mus musculus]	1.247
ENSMUSG00000 005686	Ampd3	PREDICTED: AMP deaminase 3 isoform X3 [Mus musculus]	1.220
ENSMUSG00000 026207	Speg	SPEG complex locus, isoform CRA_b, partial [Mus musculus]	1.056
ENSMUSG00000 021040	Slirp	SRA stem-loop-interacting RNA-binding protein, mitochondrial [Mus musculus]	0.884
ENSMUSG00000 030533	Unc45a	protein unc-45 homolog A [Mus musculus]	0.849
ENSMUSG00000 033701	Acbd6	acyl-CoA-binding domain-containing protein 6 isoform 1 [Mus musculus]	0.836
ENSMUSG00000 005514	Por	NADPH--cytochrome P450 reductase [Mus musculus]	0.824
ENSMUSG00000 068039	Tcp1	T-complex protein 1 subunit alpha isoform 1 [Mus musculus]	0.802
ENSMUSG00000 025613	Cct8	mKIAA0002 protein, partial [Mus musculus]	0.799
ENSMUSG00000	Csnk1g	casein kinase I isoform gamma-2 isoform	0.793

003345	2	1 [Mus musculus]	
ENSMUSG00000 020869	Lrrc59	leucine-rich repeat-containing protein 59 [Mus musculus]	0.791
ENSMUSG00000 029147	Ppm1g	protein phosphatase 1G [Mus musculus]	0.789
ENSMUSG00000 021760	Gpx8	RIKEN cDNA 2310016C16, partial [Mus musculus]	0.789
ENSMUSG00000 025232	Hexa	beta-hexosaminidase subunit alpha preproprotein [Mus musculus]	0.788
ENSMUSG00000 020844	Nxn	nucleoredoxin [Mus musculus]	0.787
ENSMUSG00000 063065	Mapk3	extracellular signal-regulated kinase 1b [Rattus norvegicus]	0.787
ENSMUSG00000 024538	Ppic	peptidyl-prolyl cis-trans isomerase C precursor [Mus musculus]	0.786
ENSMUSG00000 029209	Gnpda2	glucosamine-6-phosphate isomerase 2 isoform 1 [Mus musculus]	0.786
ENSMUSG00000 023913	Pla2g7	platelet-activating factor acetylhydrolase precursor [Mus musculus]	0.785
ENSMUSG00000 005378	Wbscr2 2	hypothetical protein [Mus musculus]	0.784
ENSMUSG00000	Nhp2l1	NHP2-like protein 1 [Mesocricetus	0.783

063480		auratus]	
ENSMUSG00000025364	Pa2g4	PREDICTED: proliferation-associated protein 2G4 [Microtus ochrogaster]	0.783
ENSMUSG00000024966	Stip1	stress-induced-phosphoprotein 1 [Mus musculus]	0.781
ENSMUSG00000024735	Prpf19	pre-mRNA-processing factor 19 isoform 1 [Mus musculus]	0.780
ENSMUSG00000025724	Sec11a	Sec11-like 1 (S. cerevisiae), isoform CRA_d [Mus musculus]	0.780
ENSMUSG00000025403	Shmt2	serine hydroxymethyltransferase, mitochondrial isoform 1 [Mus musculus]	0.780
ENSMUSG00000035960	Apex1	DNA-(apurinic or apyrimidinic site) lyase [Mus musculus]	0.779
ENSMUSG00000030890	Ilk	hypothetical protein A6R68_21216, partial [Neotoma lepida]	0.778
ENSMUSG00000062683	Atp5g2	ATP synthase F(0) complex subunit C2, mitochondrial precursor [Mus musculus]	0.777
ENSMUSG00000023110	Prmt5	protein arginine N-methyltransferase 5 isoform 1 [Mus musculus]	0.776
ENSMUSG00000066979	Bub3	mitotic checkpoint protein BUB3 isoform X1 [Ictidomys tridecemlineatus]	0.776
ENSMUSG00000	Dbi	acyl-CoA-binding protein isoform 1 [Mus	0.775

026385		musculus]	
ENSMUSG00000022024	Sugt1	protein SGT1 homolog [Mus musculus]	0.775
ENSMUSG00000025917	Cops5	COP9 signalosome complex subunit 5 isoform 1 [Mus musculus]	0.775
ENSMUSG00000030681	Mvp	major vault protein [Mus musculus]	0.774
ENSMUSG00000031672	Got2	aspartate aminotransferase, mitochondrial [Mus musculus]	0.774
ENSMUSG00000035585	Tsen34	tRNA-splicing endonuclease subunit Sen34 isoform a [Mus musculus]	0.774
ENSMUSG00000015363	Trabd	traB domain-containing protein isoform 1 [Mus musculus]	0.773
ENSMUSG00000024803	Ankrd1	ankyrin repeat domain-containing protein 1 [Mus musculus]	0.772
ENSMUSG00000040354	Mars	methionine--tRNA ligase, cytoplasmic isoform 1 [Mus musculus]	0.772
ENSMUSG00000024858	Adrbk1	beta-adrenergic receptor kinase 1 isoform 1 [Mus musculus]	0.771
ENSMUSG00000038279	Nop2	probable 28S rRNA (cytosine-C(5))-methyltransferase [Mus musculus]	0.771

ENSMUSG00000025161	Slc16a3	monocarboxylate transporter 4 [Mus caroli]	0.771
ENSMUSG00000024835	Coro1b	coronin-1B [Mus musculus]	0.770
ENSMUSG00000030751	Psma1	proteasome subunit alpha type-1 [Mus musculus]	0.769
ENSMUSG00000025225	Nfkb2	nuclear factor NF-kappa-B p100 subunit isoform a [Mus musculus]	0.769
ENSMUSG00000001380	Hars	histidine--tRNA ligase, cytoplasmic [Mus musculus]	0.768
ENSMUSG00000024997	Prdx3	thioredoxin-dependent peroxide reductase, mitochondrial precursor [Mus musculus]	0.767
ENSMUSG00000020238	Ncln	nicalin precursor [Mus musculus]	0.767
ENSMUSG00000004937	Sgta	small glutamine-rich tetratricopeptide repeat-containing protein alpha isoform 1 [Mus musculus]	0.767
ENSMUSG00000058076	Sdhc	succinate dehydrogenase cytochrome b560 subunit, mitochondrial precursor [Mus musculus]	0.767
ENSMUSG00000021957	Tkt	transketolase [Mus musculus]	0.766

ENSMUSG00000052833	Sae1	SUMO-activating enzyme subunit 1 isoform a [Mus musculus]	0.766
ENSMUSG00000005483	Dnajb1	dnaJ homolog subfamily B member 1 isoform 1 [Mus musculus]	0.766
ENSMUSG00000020706	Ftsj3	pre-rRNA processing protein FTSJ3 [Mus musculus]	0.766
ENSMUSG00000030682	Cdipt	CDP-diacylglycerol--inositol 3-phosphatidyltransferase isoform 1 [Mus musculus]	0.765
ENSMUSG00000071547	Nt5dc2	rCG42310 [Rattus norvegicus]	0.764
ENSMUSG00000039615	Stub1	STIP1 homology and U box-containing protein 1 [Mus musculus]	0.763
ENSMUSG00000034729	Mrps10	28S ribosomal protein S10, mitochondrial isoform 1 [Mus musculus]	0.763
ENSMUSG00000029017	Pmpcb	mitochondrial-processing peptidase subunit beta precursor [Mus musculus]	0.761
ENSMUSG00000033423	Eri3	ERI1 exoribonuclease 3 isoform a [Mus musculus]	0.761
ENSMUSG00000008348	Ubc	polyubiquitin-C [Mus musculus]	0.760
ENSMUSG00000	Cdc20	cell division cycle protein 20 homolog	0.760

006398		[Mus musculus]	
ENSMUSG00000026914	Psmd14	PREDICTED: 26S proteasome non-ATPase regulatory subunit 14 isoform X1 [Nannospalax galili]	0.759
ENSMUSG00000040562	Gstm2	mCG4061, partial [Mus musculus]	0.759
ENSMUSG00000011179	Odc1	ornithine decarboxylase [Mus musculus]	0.758
ENSMUSG00000027405	Nop56	nucleolar protein 56 [Mus musculus]	0.758
ENSMUSG00000020018	Snrpf	Snrpf protein, partial [Mus musculus]	0.757
ENSMUSG00000003848	Nob1	RNA-binding protein NOB1 [Mus musculus]	0.757
ENSMUSG00000060147	Serpinb6a	serine (or cysteine) peptidase inhibitor, clade B, member 6a, isoform CRA_b, partial [Mus musculus]	0.757
ENSMUSG00000022635	Zcrb1	zinc finger CCHC-type and RNA-binding motif-containing protein 1 isoform X2 [Mus pahari]	0.756
ENSMUSG00000031505	Carkd	ATP-dependent (S)-NAD(P)H-hydrate dehydratase isoform 1 [Mus musculus]	0.756

ENSMUSG00000025027	Xpnpep1	xaa-Pro aminopeptidase 1 [Mus musculus]	0.756
ENSMUSG00000021273	Fdft1	squalene synthase [Mus musculus]	0.756
ENSMUSG00000002844	Adprh	ADP-ribosylarginine hydrolase [Mus musculus]	0.756
ENSMUSG00000039682	Lap3	cytosol aminopeptidase [Mus musculus]	0.756
ENSMUSG00000025781	Atp5c1	ATP synthase subunit gamma, mitochondrial isoform a [Mus musculus]	0.756
ENSMUSG00000020440	Arf5	PREDICTED: ADP-ribosylation factor 5-like [Peromyscus maniculatus bairdii]	0.756
ENSMUSG00000030884	Uqcrc2	cytochrome b-c1 complex subunit 2, mitochondrial precursor [Mus musculus]	0.755
ENSMUSG00000018574	Acadvl	very long-chain specific acyl-CoA dehydrogenase, mitochondrial precursor [Mus musculus]	0.755
ENSMUSG00000046756	Mrps7	28S ribosomal protein S7, mitochondrial precursor [Mus musculus]	0.755
ENSMUSG00000024952	Rps6ka4	ribosomal protein S6 kinase alpha-4 [Mus musculus]	0.754
ENSMUSG00000	Psma3	proteasome subunit alpha type-3 isoform	0.754

060073		X1 [Meriones unguiculatus]	
ENSMUSG00000020321	Mdh1	malate dehydrogenase 1, NAD (soluble), isoform CRA_c, partial [Mus musculus]	0.754
ENSMUSG00000034765	Dusp5	dual specificity protein phosphatase 5 [Mus musculus]	0.754
ENSMUSG00000020386	Sar1b	GTP-binding protein SAR1b [Mus musculus]	0.754
ENSMUSG00000002332	Dhrs1	dehydrogenase/reductase SDR family member 1 [Mus musculus]	0.753
ENSMUSG00000010095	Slc3a2	4F2 cell-surface antigen heavy chain isoform a [Mus musculus]	0.753
ENSMUSG00000029762	Akr1b8	aldose reductase-related protein 2 [Mus musculus]	0.753
ENSMUSG00000000959	Oxa1l	mitochondrial inner membrane protein OXA1L [Mus musculus]	0.752
ENSMUSG00000027406	Idh3b	isocitrate dehydrogenase [NAD] subunit beta, mitochondrial [Mus musculus]	0.752
ENSMUSG00000030541	Idh2	isocitrate dehydrogenase [NADP], mitochondrial precursor [Mus musculus]	0.752
ENSMUSG00000029455	Aldh2	aldehyde dehydrogenase, mitochondrial isoform 1 precursor [Mus musculus]	0.752
ENSMUSG00000	Plrg1	pleiotropic regulator 1 [Mus musculus]	0.750

027998			
ENSMUSG00000028378	Ptgr1	prostaglandin reductase 1 [Mus musculus]	0.749
ENSMUSG00000038683	Pak1ip1	p21-activated protein kinase-interacting protein 1 [Mus musculus]	0.749
ENSMUSG00000006728	Cdk4	cyclin-dependent kinase 4 isoform 1 [Mus musculus]	0.749
ENSMUSG00000068739	Sars	serine--tRNA ligase, cytoplasmic isoform 1 [Mus musculus]	0.748
ENSMUSG00000003662	Ciao1	probable cytosolic iron-sulfur protein assembly protein CIAO1 [Mus musculus]	0.748
ENSMUSG00000053898	Ech1	delta(3,5)-Delta(2,4)-dienoyl-CoA isomerase, mitochondrial precursor [Mus musculus]	0.748
ENSMUSG00000027342	Pcna	proliferating cell nuclear antigen [Mus musculus]	0.748
ENSMUSG00000003037	Rab8a	ras-related protein Rab-8A [Mus musculus]	0.748
ENSMUSG00000031400	G6pdx	glucose-6-phosphate 1-dehydrogenase X [Mus musculus]	0.747
ENSMUSG00000034424	Gcsh	glycine cleavage system H protein, mitochondrial precursor [Mus musculus]	0.746

ENSMUSG00000026209	Dnpep	aspartyl aminopeptidase isoform a [Mus musculus]	0.746
ENSMUSG00000029599	Ddx54	ATP-dependent RNA helicase DDX54 [Mus musculus]	0.746
ENSMUSG00000015994	Fnta	protein farnesyltransferase/geranylgeranyltransferase type-1 subunit alpha [Mus musculus]	0.746
ENSMUSG00000030225	Dera	deoxyribose-phosphate aldolase [Mus musculus]	0.745
ENSMUSG00000022437	Samm50	sorting and assembly machinery component 50 homolog [Mus musculus]	0.745
ENSMUSG00000057388	Mrpl18	39S ribosomal protein L18, mitochondrial [Mus musculus]	0.745
ENSMUSG00000002064	Sdf2	stromal cell-derived factor 2 isoform 1 precursor [Mus musculus]	0.745
ENSMUSG00000032301	Psma4	proteasome subunit alpha type-4 [Mus musculus]	0.744
ENSMUSG00000020116	Pno1	RNA-binding protein PNO1 [Mus musculus]	0.744
ENSMUSG00000029446	Psph	phosphoserine phosphatase [Mus musculus]	0.744
ENSMUSG00000	Nabp2	PREDICTED: SOSS complex subunit B1	0.744

025374		isoform X2 [Nannospalax galili]	
ENSMUSG00000031948	Kars	lysine--tRNA ligase isoform 1 [Mus musculus]	0.743
ENSMUSG00000059040	Eno1b	enolase 1B, retrotransposed [Mus musculus]	0.743
ENSMUSG00000007739	Cct4	T-complex protein 1 subunit delta [Mus musculus]	0.742
ENSMUSG00000026281	Dtymk	thymidylate kinase isoform 1 [Mus musculus]	0.742
ENSMUSG00000028961	Pgd	6-phosphogluconate dehydrogenase, decarboxylating isoform 1 [Mus musculus]	0.742
ENSMUSG00000005800	Mmp8	neutrophil collagenase preproprotein [Mus musculus]	0.742
ENSMUSG00000022982	Sod1	superoxide dismutase [Cu-Zn] [Mus musculus]	0.742
ENSMUSG00000032369	Plscr1	phospholipid scramblase 1 [Mus musculus]	0.742
ENSMUSG00000096472	Cdkn2d	cyclin-dependent kinase 4 inhibitor D [Mus musculus]	0.741
ENSMUSG00000057788	Ddx49	probable ATP-dependent RNA helicase DDX49 [Mus musculus]	0.741
ENSMUSG00000	Emg1	ribosomal RNA small subunit	0.741

004268		methyltransferase NEP1 [Mus musculus]	
ENSMUSG00000075705	Msrb1	methionine-R-sulfoxide reductase B1 [Mus musculus]	0.740
ENSMUSG00000027496	Aurka	aurora kinase A isoform a [Mus musculus]	0.740
ENSMUSG00000031167	Rbm3	RNA-binding protein 3 isoform 1 [Mus musculus]	0.740
ENSMUSG00000034321	Exosc1	exosome complex component CSL4 isoform 1 [Mus musculus]	0.739
ENSMUSG00000055148	Klf2	Krueppel-like factor 2 [Mus musculus]	0.739
ENSMUSG00000022391	Rangap1	ran GTPase-activating protein 1 isoform 1 [Mus musculus]	0.738
ENSMUSG00000028953	Abcf2	ATP-binding cassette sub-family F member 2 isoform 1 [Mus musculus]	0.738
ENSMUSG00000028140	Mrpl9	39S ribosomal protein L9, mitochondrial [Mus musculus]	0.737
ENSMUSG00000014504	Srp19	signal recognition particle 19 kDa protein [Mus musculus]	0.737
ENSMUSG00000035783	Acta2	Actin, aortic smooth muscle [Fukomys damarensis]	0.737
ENSMUSG00000	Ehd1	Ehd1 protein, partial [Rattus norvegicus]	0.737

024772			
ENSMUSG00000064341	mt-Nd1	NADH dehydrogenase subunit 1 (mitochondrion) [Mus musculus]	0.737
ENSMUSG00000030298	Sec13	plasma membrane calcium-transporting ATPase 2 isoform 2 [Cricetulus griseus]	0.737
ENSMUSG00000015290	Ubl4	ubiquitin-like protein 4A [Mus musculus]	0.736
ENSMUSG00000019428	Fkbp8	peptidyl-prolyl cis-trans isomerase FKBP8 isoform a [Mus musculus]	0.735
ENSMUSG00000063694	Cycs	PREDICTED: cytochrome c, somatic [Dipodomys ordii]	0.735
ENSMUSG00000072582	Ptrh2	peptidyl-tRNA hydrolase 2, mitochondrial isoform b [Mus musculus]	0.735
ENSMUSG00000020935	Dcakd	dephospho-CoA kinase domain-containing protein [Mus musculus]	0.734
ENSMUSG00000025393	Atp5b	Atp5b protein, partial [Mus musculus]	0.734
ENSMUSG00000064358	mt-Co3	cytochrome c oxidase subunit III (mitochondrion) [Mus musculus musculus]	0.734
ENSMUSG00000058600	Rpl30	PREDICTED: 60S ribosomal protein L30-like [Peromyscus maniculatus bairdii]	0.734

ENSMUSG00000003099	Ppp5c	serine/threonine-protein phosphatase 5 [Mus musculus]	0.734
ENSMUSG00000036632	Alg5	dolichyl-phosphate beta-glucosyltransferase [Mus musculus]	0.733
ENSMUSG00000031783	Polr2c	DNA-directed RNA polymerase II subunit RPB3 [Mus musculus]	0.733
ENSMUSG00000005575	Ube2m	NEDD8-conjugating enzyme Ubc12 [Meriones unguiculatus]	0.732
ENSMUSG00000078812	Eif5a	eukaryotic translation initiation factor 5A, isoform CRA_j, partial [Mus musculus]	0.732
ENSMUSG00000041278	Ttc1	tetratricopeptide repeat protein 1 [Mus musculus]	0.731
ENSMUSG00000073982	Rhog	hypothetical protein A6R68_14799 [Neotoma lepida]	0.731
ENSMUSG00000022814	Umps	uridine 5'-monophosphate synthase isoform 1 [Mus musculus]	0.731
ENSMUSG00000024646	Cyb5a	cytochrome b5 isoform 1 [Mus musculus]	0.730
ENSMUSG00000024099	Ndufv2	NADH dehydrogenase [ubiquinone] flavoprotein 2, mitochondrial isoform 1 precursor [Mus musculus]	0.730
ENSMUSG00000	Ndufs2	NADH dehydrogenase [ubiquinone]	0.729

013593		iron-sulfur protein 2, mitochondrial precursor [Mus musculus]	
ENSMUSG00000027078	Ube2l6	ubiquitin/ISG15-conjugating enzyme E2 L6 [Mus musculus]	0.729
ENSMUSG00000058558	Rpl5	60S ribosomal protein L5 [Mus musculus]	0.729
ENSMUSG00000030357	Fkbp4	peptidyl-prolyl cis-trans isomerase FKBP4 [Mus musculus]	0.729
ENSMUSG00000020708	Psmc5	26S proteasome regulatory subunit 8 [Mus musculus]	0.728
ENSMUSG00000030272	Camk1	transcriptional adapter 3-like protein [Cricetulus griseus]	0.728
ENSMUSG00000035443	Thyn1	PREDICTED: thymocyte nuclear protein 1 isoform X1 [Mus musculus]	0.728
ENSMUSG00000030867	Plk1	serine/threonine-protein kinase PLK1 [Mus musculus]	0.728
ENSMUSG00000063888	Rpl7l1	60S ribosomal protein L7-like 1 [Mus musculus]	0.727
ENSMUSG00000039356	Exosc2	exosome complex component RRP4 [Mus musculus]	0.727
ENSMUSG00000003235	Eif2b5	translation initiation factor eIF-2B subunit epsilon [Mus musculus]	0.727

ENSMUSG00000003808	Farsa	phenylalanine--tRNA ligase alpha subunit [Mus musculus]	0.727
ENSMUSG00000019173	Rab5c	ras-related protein Rab-5C isoform 2 [Mus musculus]	0.726
ENSMUSG00000073838	Tufm	elongation factor Tu, mitochondrial isoform 1 [Mus musculus]	0.726
ENSMUSG00000031068	Glrx3	glutaredoxin-3 isoform 1 [Mus musculus]	0.726
ENSMUSG00000001403	Ube2c	ubiquitin-conjugating enzyme E2 C [Mus musculus]	0.726
ENSMUSG00000028861	Mrps15	28S ribosomal protein S15, mitochondrial precursor [Mus musculus]	0.724
ENSMUSG00000030417	Pdcd5	programmed cell death protein 5 [Mus musculus]	0.724
ENSMUSG00000024870	Rab1b	ras-related protein Rab-1B [Mus musculus]	0.724
ENSMUSG00000001416	Cct3	T-complex protein 1 subunit gamma [Mus musculus]	0.724
ENSMUSG00000041926	Rnpep	aminopeptidase B isoform 1 [Mus musculus]	0.724
ENSMUSG00000020457	Drg1	PREDICTED: developmentally-regulated GTP-binding protein 1 [Cricetulus griseus]	0.723

ENSMUSG00000030007	Cct7	T-complex protein 1 subunit eta [Mus musculus]	0.723
ENSMUSG00000031536	Polb	DNA polymerase beta [Mus musculus]	0.723
ENSMUSG00000032126	Hmbs	porphobilinogen deaminase isoform 1 [Mus musculus]	0.723
ENSMUSG00000005069	Pex5	peroxisomal targeting signal 1 receptor isoform 1 [Mus musculus]	0.723
ENSMUSG00000028692	Akr1a1	alcohol dehydrogenase [NADP(+)] [Mus musculus]	0.722
ENSMUSG00000028671	Gale	galactose-4-epimerase, UDP, isoform CRA_b, partial [Mus musculus]	0.722
ENSMUSG00000071415	Rpl23	PREDICTED: 60S ribosomal protein L23 [Microtus ochrogaster]	0.721
ENSMUSG00000019853	Hebp2	heme-binding protein 2 [Mus musculus]	0.721
ENSMUSG00000032383	Ppib	peptidyl-prolyl cis-trans isomerase B precursor [Mus musculus]	0.721
ENSMUSG00000026260	Ndufa10	NADH dehydrogenase [ubiquinone] 1 alpha subcomplex subunit 10, mitochondrial precursor [Mus musculus]	0.721
ENSMUSG00000	Cct5	mKIAA0098 protein, partial [Mus	0.720

022234		musculus]	
ENSMUSG00000 024312	Wdr46	WD repeat-containing protein 46 [Mus musculus]	0.720
ENSMUSG00000 001783	Rtcb	tRNA-splicing ligase RtcB homolog [Mus musculus]	0.720
ENSMUSG00000 028964	Park7	protein/nucleic acid deglycase DJ-1 [Mus musculus]	0.720
ENSMUSG00000 022370	Mrpl13	39S ribosomal protein L13, mitochondrial [Mus musculus]	0.720
ENSMUSG00000 049401	Ogfr	opioid growth factor receptor [Mus musculus]	0.720
ENSMUSG00000 024194	Cuta	protein CutA isoform 1 precursor [Mus musculus]	0.719
ENSMUSG00000 062054	Iah1	isoamyl acetate-hydrolyzing esterase 1 homolog [Mus musculus]	0.719
ENSMUSG00000 008373	Prpf31	U4/U6 small nuclear ribonucleoprotein Prp31 isoform 1 [Mus musculus]	0.719
ENSMUSG00000 002326	Gmpr2	GMP reductase 2 [Mus musculus]	0.719
ENSMUSG00000 005410	Mcm5	DNA replication licensing factor MCM5 [Mus musculus]	0.719
ENSMUSG00000	Myl6	myosin light polypeptide 6 isoform	0.718

090841		MLC3sm [Mus musculus]	
ENSMUSG00000028837	Psmb2	proteasome subunit beta type-2 [Mus musculus]	0.718
ENSMUSG00000034875	Nudt19	nucleoside diphosphate-linked moiety X motif 19 precursor [Mus musculus]	0.718
ENSMUSG00000028691	Prdx1	peroxiredoxin-1 [Mus musculus]	0.718
ENSMUSG00000023456	Tpi1	triosephosphate isomerase [Mus musculus]	0.717
ENSMUSG00000028937	Acot7	cytosolic acyl coenzyme A thioester hydrolase isoform 1 [Mus musculus]	0.717
ENSMUSG00000022221	Ripk3	receptor-interacting serine/threonine-protein kinase 3 isoform 1 [Mus musculus]	0.716
ENSMUSG00000060636	Rpl35a	60S ribosomal protein L35a [Rattus norvegicus]	0.716
ENSMUSG00000025465	Echs1	enoyl-CoA hydratase, mitochondrial precursor [Mus musculus]	0.716
ENSMUSG00000001289	Pfdn5	prefoldin subunit 5 [Mus musculus]	0.716
ENSMUSG00000029703	Lrwd1	RIKEN cDNA 1200011O22, isoform CRA_a, partial [Mus musculus]	0.716

ENSMUSG00000029145	Eif2b4	translation initiation factor eIF-2B subunit delta isoform 2 [Mus musculus]	0.716
ENSMUSG00000037580	Gch1	GTP cyclohydrolase 1 precursor [Mus musculus]	0.716
ENSMUSG00000028597	Gpx7	glutathione peroxidase 7 precursor [Mus musculus]	0.715
ENSMUSG00000030060	Hmces	RIKEN cDNA 8430410A17, isoform CRA_b, partial [Mus musculus]	0.715
ENSMUSG00000025260	Hsd17b10	3-hydroxyacyl-CoA dehydrogenase type-2 [Mus musculus]	0.715
ENSMUSG00000062382	Gm10116	ferritin light chain 1 [Mus musculus]	0.715
ENSMUSG00000002963	Pnkp	PREDICTED: bifunctional polynucleotide phosphatase/kinase isoform X5 [Mus musculus]	0.715
ENSMUSG00000002524	Puf60	poly(U)-binding-splicing factor PUF60 isoform a [Mus musculus]	0.714
ENSMUSG00000050370	Ch25h	cholesterol 25-hydroxylase [Mus musculus]	0.714
ENSMUSG00000025872	Thoc3	THO complex subunit 3 [Mus caroli]	0.714
ENSMUSG00000	Mrpl22	PREDICTED: 39S ribosomal protein L22,	0.714

020514		mitochondrial isoform X1 [Mus musculus]	
ENSMUSG00000 029993	Nfu1	NFU1 iron-sulfur cluster scaffold homolog, mitochondrial isoform 1 precursor [Mus musculus]	0.714
ENSMUSG00000 026279	Thap4	THAP domain-containing protein 4 isoform 1 [Mus musculus]	0.713
ENSMUSG00000 069020	Urm1	ubiquitin-related modifier 1 [Mus musculus]	0.713
ENSMUSG00000 032786	Alas1	5-aminolevulinate synthase, nonspecific, mitochondrial precursor [Mus musculus]	0.713
ENSMUSG00000 020537	Drg2	developmentally-regulated GTP-binding protein 2 [Mus musculus]	0.713
ENSMUSG00000 026701	Prdx6	mKIAA0106 protein, partial [Mus musculus]	0.712
ENSMUSG00000 071662	Polr2g	PREDICTED: DNA-directed RNA polymerase II subunit RPB7 isoform X1 [Cricetulus griseus]	0.712
ENSMUSG00000 067713	Prkag1	5'-AMP-activated protein kinase subunit gamma-1 [Mus musculus]	0.712
ENSMUSG00000 021871	Pnp	purine nucleoside phosphorylase [Mus musculus]	0.711
ENSMUSG00000	Gsto1	glutathione S-transferase omega-1 [Mus	0.711

025068		musculus]	
ENSMUSG00000 019942	Cdk1	cyclin-dependent kinase 1 [Mus musculus]	0.711
ENSMUSG00000 025035	Arl3	ADP-ribosylation factor-like protein 3 isoform 1 [Mus musculus]	0.710
ENSMUSG00000 028138	Adh5	alcohol dehydrogenase class-3 isoform 1 [Mus musculus]	0.708
ENSMUSG00000 033917	Gde1	glycerophosphodiester phosphodiesterase 1 [Mus musculus]	0.708
ENSMUSG00000 026526	Fh1	fumarate hydratase, mitochondrial precursor [Mus musculus]	0.707
ENSMUSG00000 004996	Mri1	methylthioribose-1-phosphate isomerase [Mus musculus]	0.707
ENSMUSG00000 018770	Atp5g3	ATP synthase F(0) complex subunit C3, mitochondrial isoform a precursor [Mus musculus]	0.706
ENSMUSG00000 026922	Agpat2	1-acyl-sn-glycerol-3-phosphate acyltransferase beta precursor [Mus musculus]	0.706
ENSMUSG00000 033845	Mrpl15	39S ribosomal protein L15, mitochondrial isoform 1 precursor [Mus musculus]	0.706
ENSMUSG00000	Eef1g	speckle targeted PIP5K1A-regulated	0.706

071644		poly(A) polymerase isoform X2 [Ictidomys tridecemlineatus]	
ENSMUSG00000039660	D2Wsu81e	putative methyltransferase C9orf114 homolog [Mus musculus]	0.705
ENSMUSG00000040385	Ppp1ca	serine/threonine-protein phosphatase PP1-alpha catalytic subunit [Mus musculus]	0.705
ENSMUSG00000060288	Ppih	peptidyl-prolyl cis-trans isomerase H isoform 2 [Mus musculus]	0.705
ENSMUSG00000028070	Apoa1bp	NAD(P)H-hydrate epimerase precursor [Mus musculus]	0.705
ENSMUSG00000038489	Polr2l	Ab1-108 [Rattus norvegicus]	0.704
ENSMUSG00000060098	Prmt7	mKIAA1933 protein, partial [Mus musculus]	0.704
ENSMUSG00000037916	Ndufv1	NADH dehydrogenase [ubiquinone] flavoprotein 1, mitochondrial precursor [Mus musculus]	0.704
ENSMUSG00000027597	Ahcy	mCG21063, isoform CRA_b, partial [Mus musculus]	0.704
ENSMUSG00000061979	Wbscr16	RCC1-like G exchanging factor-like protein [Mus musculus]	0.704

ENSMUSG00000103472	Pcdhga7	protocadherin gamma-A7 precursor [Mus musculus]	0.704
ENSMUSG00000032306	Mpi	mannose-6-phosphate isomerase [Mus musculus]	0.704
ENSMUSG00000028673	Fuca1	tissue alpha-L-fucosidase precursor [Mus musculus]	0.703
ENSMUSG00000025290	Rps24	40S ribosomal protein S24 isoform X1 [Heterocephalus glaber]	0.703
ENSMUSG00000002767	Mrpl2	39S ribosomal protein L2, mitochondrial isoform 1 precursor [Mus musculus]	0.702
ENSMUSG00000024164	C3	complement C3 preproprotein [Mus musculus]	0.702
ENSMUSG00000025747	Tyms	thymidylate synthase, isoform CRA_a, partial [Mus musculus]	0.702
ENSMUSG00000050335	Lgals3	galectin-3 [Mus musculus]	0.701
ENSMUSG00000030826	Bcat2	branched-chain-amino-acid aminotransferase, mitochondrial isoform 1 precursor [Mus musculus]	0.701
ENSMUSG00000049960	Mrps16	28S ribosomal protein S16, mitochondrial precursor [Mus musculus]	0.701
ENSMUSG00000	Ethe1	persulfide dioxygenase ETHE1,	0.700

064254		mitochondrial precursor [Mus musculus]	
ENSMUSG00000079641	Rpl39	mCG146274, partial [Mus musculus]	0.700
ENSMUSG00000028772	Zcchc17	nucleolar protein of 40 kDa isoform X1 [Mus pahari]	0.700
ENSMUSG00000028334	Nans	sialic acid synthase [Mus musculus]	0.699
ENSMUSG00000007041	Clic1	chloride intracellular channel protein 1 [Mus musculus]	0.699
ENSMUSG00000029713	Gnb2	guanine nucleotide-binding protein G(I)/G(S)/G(T) subunit beta-2 [Mus musculus]	0.699
ENSMUSG00000036613	Tssc1	EARP-interacting protein [Mus musculus]	0.698
ENSMUSG00000016503	Gtf3a	general transcription factor III A, isoform CRA_a, partial [Mus musculus]	0.698
ENSMUSG00000070284	Gmppb	PREDICTED: mannose-1-phosphate guanyltransferase beta isoform X1 [Rattus norvegicus]	0.698
ENSMUSG00000006818	Sod2	manganese superoxide dismutase [Mus musculus]	0.698
ENSMUSG00000	Fkbp2	peptidyl-prolyl cis-trans isomerase FKBP2	0.697

056629		isoform X1 [Mus pahari]	
ENSMUSG00000 034974	Dapk3	PREDICTED: death-associated protein kinase 3 isoform X1 [Rattus norvegicus]	0.697
ENSMUSG00000 044303	Cdkn2a	cyclin-dependent kinase inhibitor 2A p16INK4a [Mus musculus]	0.696
ENSMUSG00000 073702	Rpl31	mCG126194, isoform CRA_b [Mus musculus]	0.695
ENSMUSG00000 021024	Psma6	proteasome (prosome, macropain) subunit, alpha type 6, isoform CRA_b, partial [Mus musculus]	0.695
ENSMUSG00000 042660	Wdr55	WD repeat-containing protein 55 [Mus musculus]	0.695
ENSMUSG00000 028029	Aimp1	aminoacyl tRNA synthase complex-interacting multifunctional protein 1 [Mus musculus]	0.695
ENSMUSG00000 007950	Abhd8	protein ABHD8 [Mus musculus]	0.695
ENSMUSG00000 020225	Tmbim4	protein lifeguard 4 [Mus musculus]	0.694
ENSMUSG00000 049751	Rpl36al	LOW QUALITY PROTEIN: 60S ribosomal protein L36a [Mus caroli]	0.694
ENSMUSG00000	Atp5o	ATP synthase subunit O, mitochondrial	0.694

022956		precursor [Mus musculus]	
ENSMUSG00000039183	Nubp2	cytosolic Fe-S cluster assembly factor NUBP2 isoform 1 [Mus musculus]	0.693
ENSMUSG00000035493	Tgfbi	transforming growth factor-beta-induced protein ig-h3 precursor [Mus musculus]	0.693
ENSMUSG00000025289	Prdx4	peroxiredoxin-4 isoform 2 precursor [Mus musculus]	0.693
ENSMUSG00000030079	Ruvbl1	ruvB-like 1 [Mus musculus]	0.693
ENSMUSG00000060803	Gstp1	glutathione S-transferase P 1 [Mus musculus]	0.693
ENSMUSG00000029622	Arpc1b	actin-related protein 2/3 complex subunit 1B [Mus musculus]	0.693
ENSMUSG00000041506	Rrp9	U3 small nucleolar RNA-interacting protein 2 [Mus musculus]	0.693
ENSMUSG00000021996	Esd	S-formylglutathione hydrolase isoform 1 [Mus musculus]	0.693
ENSMUSG00000032399	Rpl4	PREDICTED: 60S ribosomal protein L4 [Dipodomys ordii]	0.692
ENSMUSG00000078440	Dohh	deoxyhypusine hydroxylase [Mus musculus]	0.692
ENSMUSG00000	Psmb1	proteasome subunit beta type-1 precursor	0.691

014769		[Mus musculus]	
ENSMUSG00000029028	Lrrc47	leucine-rich repeat-containing protein 47 [Mus musculus]	0.690
ENSMUSG00000000399	Ndufa9	NADH dehydrogenase [ubiquinone] 1 alpha subcomplex subunit 9, mitochondrial precursor [Mus musculus]	0.690
ENSMUSG00000030603	Psmc4	26S proteasome regulatory subunit 6B [Rattus norvegicus]	0.690
ENSMUSG00000038462	Uqcrfs1	cytochrome b-c1 complex subunit Rieske, mitochondrial [Mus musculus]	0.690
ENSMUSG00000070953	Rabepk	rab9 effector protein with kelch motifs [Mus musculus]	0.690
ENSMUSG00000029642	Polr1d	DNA-directed RNA polymerases I and III subunit RPAC2 isoform 1 [Mus musculus]	0.689
ENSMUSG00000024644	Cndp2	cytosolic non-specific dipeptidase [Mus musculus]	0.689
ENSMUSG00000025503	Taldo1	transaldolase [Mus musculus]	0.689
ENSMUSG00000028443	Nudt2	Nudt2 gene product [Mus musculus]	0.689
ENSMUSG00000019139	Isyna1	inositol-3-phosphate synthase 1 [Mus musculus]	0.688

ENSMUSG00000002233	Rhoc	p21RhoC [Mus musculus]	0.688
ENSMUSG00000015671	Psma2	proteasome subunit alpha type-2 [Rattus norvegicus]	0.687
ENSMUSG00000034892	Rps29	mCG7602, partial [Mus musculus]	0.687
ENSMUSG00000030595	Nfkbib	NF-kappa-B inhibitor beta [Mus musculus]	0.686
ENSMUSG00000028898	Trnau1ap	tRNA selenocysteine 1-associated protein 1 [Mus musculus]	0.686
ENSMUSG00000041571	Sepw1	selenoprotein W [Mus caroli]	0.686
ENSMUSG00000016256	Ctsz	cathepsin Z preproprotein [Mus musculus]	0.686
ENSMUSG00000028932	Psmc2	26S proteasome regulatory subunit 7 [Mus musculus]	0.686
ENSMUSG00000042729	Wdr74	WD repeat-containing protein 74 [Mus musculus]	0.686
ENSMUSG00000021178	Psmc1	26S protease regulatory subunit 4 [Heterocephalus glaber]	0.685
ENSMUSG00000052456	Asna1	PREDICTED: ATPase ASNA1 [Nannospalax galili]	0.685

ENSMUSG00000079435	Rpl36a	LOW QUALITY PROTEIN: 60S ribosomal protein L36a [Mus caroli]	0.684
ENSMUSG00000053398	Phgdh	D-3-phosphoglycerate dehydrogenase [Mus musculus]	0.684
ENSMUSG00000026377	Nifk	MKI67 FHA domain-interacting nucleolar phosphoprotein [Mus musculus]	0.681
ENSMUSG00000031429	Psmd10	26S proteasome non-ATPase regulatory subunit 10 isoform 1 [Mus musculus]	0.680
ENSMUSG00000068749	Psma5	proteasome subunit alpha type-5 [Mus musculus]	0.680
ENSMUSG00000062345	Serpinb2	PREDICTED: plasminogen activator inhibitor 2, macrophage isoform X1 [Mus musculus]	0.680
ENSMUSG00000003299	Mrpl4	Mrpl4 protein, partial [Mus musculus]	0.680
ENSMUSG00000026750	Psmb7	proteasome subunit beta type-7 precursor [Mus musculus]	0.680
ENSMUSG00000060376	Bckdha	2-oxoisovalerate dehydrogenase subunit alpha, mitochondrial [Mus musculus]	0.680
ENSMUSG00000020267	Hint1	histidine triad nucleotide-binding protein 1 [Mus musculus]	0.679
ENSMUSG00000	Hspe1	10 kDa heat shock protein, mitochondrial	0.679

073676		[Mus musculus]	
ENSMUSG00000 020089	Ppa1	pyrophosphatase (inorganic) 1, partial [Mus musculus]	0.679
ENSMUSG00000 068417	Pnp2	purine-nucleoside phosphorylase 2 [Mus musculus]	0.679
ENSMUSG00000 003868	Ruvbl2	ruvB-like 2 [Mus musculus]	0.678
ENSMUSG00000 022474	Pmm1	phosphomannomutase 1 isoform 1 [Mus musculus]	0.678
ENSMUSG00000 005803	Sqrdl	sulfide:quinone oxidoreductase, mitochondrial [Mus musculus]	0.678
ENSMUSG00000 059323	Tonsl	tonsoku-like protein [Mus musculus]	0.677
ENSMUSG00000 024436	Mrps18 b	28S ribosomal protein S18b, mitochondrial isoform 1 [Mus musculus]	0.676
ENSMUSG00000 061787	Rps17	40S ribosomal protein S17 [Mus musculus]	0.676
ENSMUSG00000 026956	Uap1l1	UDP-N-acetylhexosamine pyrophosphorylase-like protein 1 [Mus musculus]	0.675
ENSMUSG00000 046865	Fbl	PREDICTED: rRNA 2'-O-methyltransferase fibrillarin	0.675

		[Microtus ochrogaster]	
ENSMUSG00000 002580	Mien1	migration and invasion enhancer 1 [Mus musculus]	0.675
ENSMUSG00000 028848	Gpn2	GPN-loop GTPase 2 isoform 1 [Mus musculus]	0.675
ENSMUSG00000 062867	Impdh2	inosine-5'-monophosphate dehydrogenase 2 [Mus musculus]	0.674
ENSMUSG00000 026820	Ptges2	prostaglandin E synthase 2 [Mus musculus]	0.674
ENSMUSG00000 020444	Guk1	guanylate kinase isoform 1 [Mus musculus]	0.674
ENSMUSG00000 078695	Cisd3	CDGSH iron-sulfur domain-containing protein 3, mitochondrial precursor [Mus musculus]	0.673
ENSMUSG00000 095687	Rnaset2 a	ribonuclease T2-B precursor [Mus musculus]	0.673
ENSMUSG00000 030189	Ybx3	Y-box-binding protein 3 long isoform [Mus musculus]	0.672
ENSMUSG00000 005447	Pafah1b 3	platelet-activating factor acetylhydrolase IB subunit gamma [Mus musculus]	0.672
ENSMUSG00000 098274	Rpl24	Rpl24 protein, partial [Mus musculus]	0.671

ENSMUSG00000006299	Aamp	angio-associated migratory cell protein isoform 1 [Mus musculus]	0.671
ENSMUSG00000004929	Thop1	thimet oligopeptidase [Mus musculus]	0.671
ENSMUSG00000019179	Mdh2	malate dehydrogenase, mitochondrial precursor [Mus musculus]	0.670
ENSMUSG00000000295	Hddc2	HD domain-containing protein 2 [Mus musculus]	0.669
ENSMUSG00000002102	Psmc3	proteasome (prosome, macropain) 26S subunit, ATPase 3, isoform CRA_c [Mus musculus]	0.668
ENSMUSG00000035754	Wdr18	WD repeat-containing protein 18 [Mus musculus]	0.668
ENSMUSG00000035027	Map2k2	dual specificity mitogen-activated protein kinase kinase 2 isoform 1 [Mus musculus]	0.668
ENSMUSG00000029066	Mrpl20	39S ribosomal protein L20, mitochondrial precursor [Mus musculus]	0.668
ENSMUSG00000018858	Ict1	peptidyl-tRNA hydrolase ICT1, mitochondrial isoform 2 precursor [Mus musculus]	0.667
ENSMUSG00000025825	Iscu	mCG3543, isoform CRA_a [Mus musculus]	0.667

ENSMUSG00000019505	Ubb	polyubiquitin-B [Mus musculus]	0.666
ENSMUSG00000057841	Rpl32	60S ribosomal protein L32 [Mesocricetus auratus]	0.666
ENSMUSG00000004610	Etfb	electron transfer flavoprotein subunit beta [Mus musculus]	0.665
ENSMUSG00000028495	Rps6	40S ribosomal protein S6 [Mus musculus]	0.664
ENSMUSG00000020736	Nt5c	5'(3')-deoxyribonucleotidase, cytosolic type [Mus musculus]	0.664
ENSMUSG00000008683	Rps15a	Rps15a protein, partial [Mus musculus]	0.664
ENSMUSG00000031383	Dusp9	dual specificity phosphatase 9, isoform CRA_c [Mus musculus]	0.663
ENSMUSG00000070394	Tmem256	transmembrane protein 256 precursor [Mus musculus]	0.663
ENSMUSG00000022503	Nubp1	cytosolic Fe-S cluster assembly factor NUBP1 [Mus musculus]	0.663
ENSMUSG00000006057	Atp5g1	ATP synthase F(0) complex subunit C1, mitochondrial [Mus musculus]	0.663
ENSMUSG00000022407	Adsl	adenylosuccinate lyase [Mus musculus]	0.663

ENSMUSG00000 024158	Hagh	hydroxyacylglutathione hydrolase, mitochondrial isoform 1 precursor [Mus musculus]	0.662
ENSMUSG00000 046364	Rpl27a	60S ribosomal protein L27a [Mesocricetus auratus]	0.661
ENSMUSG00000 004393	Ddx56	probable ATP-dependent RNA helicase DDX56 [Mus musculus]	0.661
ENSMUSG00000 022557	Bop1	ribosome biogenesis protein BOP1 [Mus musculus]	0.660
ENSMUSG00000 012405	Rpl15	60S ribosomal protein L15 [Mus musculus]	0.660
ENSMUSG00000 040688	Tbl3	transducin beta-like protein 3 [Mus musculus]	0.660
ENSMUSG00000 010911	Apip	methylthioribulose-1-phosphate dehydratase isoform 1 [Mus musculus]	0.660
ENSMUSG00000 059743	Fdps	farnesyl pyrophosphate synthase isoform 2 [Mus musculus]	0.660
ENSMUSG00000 006517	Mvd	diphosphomevalonate decarboxylase [Mus musculus]	0.659
ENSMUSG00000 025794	Rpl14	60S ribosomal protein L14 [Mus musculus]	0.659
ENSMUSG00000	Mif	macrophage migration inhibitory factor	0.659

033307		[Mus musculus]	
ENSMUSG00000 028367	Txn1	thioredoxin [Mus musculus]	0.659
ENSMUSG00000 047215	Rpl9	60S ribosomal protein L9 [Mus musculus]	0.659
ENSMUSG00000 041841	Rpl37	PREDICTED: 60S ribosomal protein L37 [Nannospalax galili]	0.659
ENSMUSG00000 005510	Ndufs3	NADH dehydrogenase [ubiquinone] iron-sulfur protein 3, mitochondrial precursor [Mus musculus]	0.658
ENSMUSG00000 006378	Gcat	2-amino-3-ketobutyrate coenzyme A ligase, mitochondrial isoform a [Mus musculus]	0.658
ENSMUSG00000 026036	Nif3l1	NIF3-like protein 1 [Mus musculus]	0.658
ENSMUSG00000 025651	Uqcrc1	cytochrome b-c1 complex subunit 1, mitochondrial precursor [Mus musculus]	0.658
ENSMUSG00000 067288	Rps28	ribosomal protein S28, partial [Cricetulus griseus]	0.657
ENSMUSG00000 031388	Naa10	N-alpha-acetyltransferase 10 isoform 1 [Mus musculus]	0.657
ENSMUSG00000	Prdx2	peroxiredoxin-2 [Mus musculus]	0.657

005161			
ENSMUSG00000040034	Nup43	nucleoporin 43, isoform CRA_a, partial [Mus musculus]	0.657
ENSMUSG00000024132	Eci1	enoyl-CoA delta isomerase 1, mitochondrial precursor [Mus musculus]	0.656
ENSMUSG00000006717	Acot13	acyl-coenzyme A thioesterase 13 [Mus musculus]	0.656
ENSMUSG00000050708	Ftl1	ferritin light chain 1 [Mus musculus]	0.655
ENSMUSG00000036880	Acaa2	3-ketoacyl-CoA thiolase, mitochondrial [Mus musculus]	0.655
ENSMUSG00000067274	Rplp0	60S acidic ribosomal protein P0 [Mus musculus]	0.654
ENSMUSG00000070319	Eif3g	eukaryotic translation initiation factor 3 subunit G [Mus musculus]	0.654
ENSMUSG00000015806	Qdpr	dihydropteridine reductase [Mus musculus]	0.654
ENSMUSG00000048755	Mcat	malonyl-CoA-acyl carrier protein transacylase, mitochondrial [Mus musculus]	0.653
ENSMUSG00000022013	Dnajc15	dnaJ homolog subfamily C member 15 [Mus musculus]	0.653

ENSMUSG00000028684	Urod	uroporphyrinogen decarboxylase [Mus musculus]	0.652
ENSMUSG00000022570	Tsta3	tissue specific transplantation antigen P35B, isoform CRA_b, partial [Mus musculus]	0.652
ENSMUSG00000028798	Eif3i	eukaryotic translation initiation factor 3 subunit I [Mus musculus]	0.652
ENSMUSG00000003429	Rps11	PREDICTED: 40S ribosomal protein S11 [Dipodomys ordii]	0.652
ENSMUSG00000029198	Grpel1	grpE protein homolog 1, mitochondrial [Mus caroli]	0.652
ENSMUSG00000059291	Rpl11	60S ribosomal protein L11 [Mus musculus]	0.652
ENSMUSG00000022193	Psmb5	proteasome subunit beta type-5 [Mus musculus]	0.651
ENSMUSG00000067148	Polr1c	DNA-directed RNA polymerases I and III subunit RPAC1 [Mus musculus]	0.651
ENSMUSG00000020471	Pold2	DNA polymerase delta subunit 2 [Mus musculus]	0.651
ENSMUSG00000030879	Mrpl17	39S ribosomal protein L17, mitochondrial [Mus caroli]	0.650
ENSMUSG00000	Rpl34	60S ribosomal protein L34 [Mus	0.649

062006		musculus]	
ENSMUSG00000079677	Fdx1l	ferredoxin-2, mitochondrial precursor [Mus musculus]	0.649
ENSMUSG00000006442	Srm	spermidine synthase [Mus musculus]	0.649
ENSMUSG00000027384	Ndufaf5	arginine-hydroxylase NDUFAF5, mitochondrial precursor [Mus musculus]	0.648
ENSMUSG00000028081	Rps3a1	40S ribosomal protein S3a [Mus musculus]	0.648
ENSMUSG00000001270	Ckb	creatine kinase B-type [Mus musculus]	0.647
ENSMUSG00000058267	Mrps14	28S ribosomal protein S14, mitochondrial isoform 1 [Mus musculus]	0.647
ENSMUSG00000038387	Rras	ras-related protein R-Ras isoform 1 precursor [Mus musculus]	0.647
ENSMUSG00000045948	Mrps12	28S ribosomal protein S12, mitochondrial isoform a precursor [Mus musculus]	0.647
ENSMUSG00000021591	Glrx	glutaredoxin-1 [Mus musculus]	0.647
ENSMUSG00000004285	Atp6v1f	V-type proton ATPase subunit F [Rattus norvegicus]	0.646
ENSMUSG00000	Mrpl36	39S ribosomal protein L36, mitochondrial	0.646

021607		[Mus musculus]	
ENSMUSG00000036781	Rps27l	40S ribosomal protein S27-like isoform 1 [Mus musculus]	0.645
ENSMUSG00000030538	Cib1	calcium and integrin-binding protein 1 isoform 1 [Mus musculus]	0.644
ENSMUSG00000008668	Rps18	mCG114988 [Mus musculus]	0.644
ENSMUSG00000059734	Ndufs8	NADH dehydrogenase [ubiquinone] iron-sulfur protein 8, mitochondrial [Mus musculus]	0.644
ENSMUSG00000090733	Rps27	PREDICTED: 40S ribosomal protein S27-like [Microtus ochrogaster]	0.644
ENSMUSG00000020857	Nme2	nucleoside diphosphate kinase B [Mus musculus]	0.643
ENSMUSG00000001642	Akr1b3	aldose reductase [Mus musculus]	0.642
ENSMUSG00000021025	Nfkbia	nuclear factor of kappa light chain gene enhancer in B-cells inhibitor, alpha, partial [Mus musculus]	0.642
ENSMUSG00000025580	Eif4a3	eukaryotic initiation factor 4A-III [Mus musculus]	0.642
ENSMUSG00000	Grhpr	glyoxylate reductase/hydroxypyruvate	0.642

035637		reductase isoform 1 [Mus musculus]	
ENSMUSG00000004264	Phb2	prohibitin-2 [Mus musculus]	0.642
ENSMUSG00000032171	Pin1	peptidyl-prolyl cis-trans isomerase NIMA-interacting 1 [Mus musculus]	0.642
ENSMUSG00000023967	Mrps18a	28S ribosomal protein S18a, mitochondrial precursor [Mus musculus]	0.641
ENSMUSG00000029545	Acads	short-chain specific acyl-CoA dehydrogenase, mitochondrial [Mus pahari]	0.641
ENSMUSG00000028651	Ppie	peptidyl-prolyl cis-trans isomerase E [Mus musculus]	0.641
ENSMUSG00000038650	Rnh1	ribonuclease inhibitor isoform a [Mus musculus]	0.640
ENSMUSG00000028741	Mrto4	mRNA turnover protein 4 homolog isoform 1 [Mus musculus]	0.640
ENSMUSG00000001285	Myg1	UPF0160 protein MYG1, mitochondrial precursor [Mus musculus]	0.640
ENSMUSG00000004667	Polr2e	DNA-directed RNA polymerases I, II, and III subunit RPABC1 [Mus musculus]	0.639
ENSMUSG00000063457	Rps15	40S ribosomal protein S15 [Ictidomys tridecemlineatus]	0.639

ENSMUSG00000002010	Idh3g	isocitrate dehydrogenase [NAD] subunit gamma 1, mitochondrial isoform 1 precursor [Mus musculus]	0.638
ENSMUSG00000025362	Rps26	40S ribosomal protein S26 [Rattus norvegicus]	0.638
ENSMUSG00000011254	Thg1l	probable tRNA(His) guanylyltransferase isoform 1 [Mus musculus]	0.638
ENSMUSG00000002797	Ggct	gamma-glutamylcyclotransferase [Mus musculus]	0.638
ENSMUSG00000028910	Mecr	enoyl-[acyl-carrier-protein] reductase, mitochondrial precursor [Mus musculus]	0.638
ENSMUSG00000015092	Edf1	endothelial differentiation-related factor 1 [Mus musculus]	0.638
ENSMUSG00000060938	Rpl26	PREDICTED: 60S ribosomal protein L26-like [Microtus ochrogaster]	0.637
ENSMUSG00000021606	Ndufs6	NADH dehydrogenase [ubiquinone] iron-sulfur protein 6, mitochondrial precursor [Mus musculus]	0.637
ENSMUSG00000021102	Glrx5	glutaredoxin-related protein 5, mitochondrial [Mus caroli]	0.637
ENSMUSG00000058546	Rpl23a	PREDICTED: 60S ribosomal protein L23a isoform X1 [Cricetulus griseus]	0.636

ENSMUSG00000030652	Coq7	5-demethoxyubiquinone hydroxylase, mitochondrial isoform 2 [Mus musculus]	0.636
ENSMUSG00000020372	Gnb2l1	protein kinase C receptor [Rattus norvegicus]	0.636
ENSMUSG00000024121	Atp6v0c	V-type proton ATPase 16 kDa proteolipid subunit [Mus musculus]	0.636
ENSMUSG00000008682	Rpl10	60S ribosomal protein L10 [Rattus norvegicus]	0.636
ENSMUSG00000074797	Itpa	inosine triphosphate pyrophosphatase [Mus musculus]	0.634
ENSMUSG00000027566	Psma7	proteasome subunit alpha type-7 isoform 1 [Mus musculus]	0.633
ENSMUSG00000038845	Phb	prohibitin [Mus musculus]	0.633
ENSMUSG00000034880	Mrpl34	39S ribosomal protein L34, mitochondrial [Mus musculus]	0.633
ENSMUSG00000022551	Cyc1	cytochrome c1, heme protein, mitochondrial [Mus musculus]	0.632
ENSMUSG00000069744	Psmb3	proteasome subunit beta type-3 [Mus musculus]	0.631
ENSMUSG00000035215	Lsm7	U6 snRNA-associated Sm-like protein LSm7 isoform 4 [Mus musculus]	0.630

ENSMUSG00000032518	Rpsa	mCG2650 [Mus musculus]	0.629
ENSMUSG00000020766	Galk1	Galactokinase 1 [Mus musculus]	0.629
ENSMUSG00000024414	Mrpl27	39S ribosomal protein L27, mitochondrial [Mus musculus]	0.628
ENSMUSG00000038900	Rpl12	60S ribosomal protein L12 [Mus musculus]	0.628
ENSMUSG00000042462	Dctpp1	dCTP pyrophosphatase 1 [Mus musculus]	0.628
ENSMUSG00000026520	Pycr2	pyrroline-5-carboxylate reductase 2 [Mus musculus]	0.627
ENSMUSG00000028743	Akr7a5	aflatoxin B1 aldehyde reductase member 2 [Mus musculus]	0.626
ENSMUSG00000024661	Fth1	ferritin heavy chain [Mus musculus]	0.625
ENSMUSG00000078931	Pdf	peptide deformylase, mitochondrial [Mus musculus]	0.625
ENSMUSG00000063931	Pepd	xaa-Pro dipeptidase [Mus musculus]	0.624
ENSMUSG00000000740	Rpl13	60S ribosomal protein L13 [Mus musculus]	0.624

ENSMUSG00000047675	Rps8	40S ribosomal protein S8 [Ictidomys tridecemlineatus]	0.622
ENSMUSG00000054716	Zfp771	zinc finger protein 771 isoform X2 [Mesocricetus auratus]	0.622
ENSMUSG00000026177	Slc11a1	natural resistance-associated macrophage protein 1 [Mus musculus]	0.621
ENSMUSG00000003072	Atp5d	ATP synthase subunit delta, mitochondrial isoform 1 precursor [Mus musculus]	0.620
ENSMUSG00000090862	Rps13	PREDICTED: 40S ribosomal protein S13 [Dipodomys ordii]	0.620
ENSMUSG00000063856	Gpx1	glutathione peroxidase 1 isoform 1 [Mus musculus]	0.619
ENSMUSG00000036733	Rbm42	RNA-binding protein 42 isoform X3 [Heterocephalus glaber]	0.619
ENSMUSG00000023939	Mrpl14	39S ribosomal protein L14, mitochondrial precursor [Mus musculus]	0.618
ENSMUSG00000024944	Arl2	ADP-ribosylation factor-like protein 2 [Mus musculus]	0.617
ENSMUSG00000006589	Aprt	adenine phosphoribosyltransferase [Mus musculus]	0.615
ENSMUSG00000043445	Pgp	glycerol-3-phosphate phosphatase [Mus musculus]	0.615

ENSMUSG00000022947	Cbr3	carbonyl reductase [NADPH] 3 [Mus musculus]	0.614
ENSMUSG00000031171	Ftsj1	putative tRNA (cytidine(32)/guanosine(34)-2'-O)-methyltransferase isoform 1 [Mus musculus]	0.614
ENSMUSG00000003355	Fkbp11	peptidyl-prolyl cis-trans isomerase FKBP11 isoform 1 precursor [Mus musculus]	0.614
ENSMUSG00000052926	Rnaseh2a	ribonuclease H2 subunit A [Mus musculus]	0.614
ENSMUSG00000053746	Ptrh1	probable peptidyl-tRNA hydrolase [Mus musculus]	0.613
ENSMUSG00000032673	Prorsd1	prolyl-tRNA synthetase associated domain-containing protein 1 isoform 2 [Mus musculus]	0.612
ENSMUSG00000070283	Ndufaf3	NADH dehydrogenase [ubiquinone] 1 alpha subcomplex assembly factor 3 [Mus musculus]	0.612
ENSMUSG00000053801	Grwd1	glutamate-rich WD repeat-containing protein 1 [Mus musculus]	0.611
ENSMUSG00000	Sco2	protein SCO2 homolog, mitochondrial	0.611

091780		[Mus musculus]	
ENSMUSG00000063171	Rps4l	40S ribosomal protein S4, X isoform-like [Mus caroli]	0.611
ENSMUSG00000024338	Psmb8	proteasome subunit beta type-8 precursor [Mus musculus]	0.611
ENSMUSG00000037563	Rps16	Rps16 protein, partial [Rattus norvegicus]	0.611
ENSMUSG00000052429	Prmt1	protein arginine N-methyltransferase 1 isoform 1 [Mus musculus]	0.610
ENSMUSG00000030148	Clec4a2	C-type lectin domain family 4 member A isoform a [Mus musculus]	0.610
ENSMUSG00000010376	Nedd8	PREDICTED: NEDD8 [Jaculus jaculus]	0.610
ENSMUSG00000041845	Rhod	rho-related GTP-binding protein RhoD 1 [Mus musculus]	0.610
ENSMUSG00000022126	Irg1	mCG114119, partial [Mus musculus]	0.609
ENSMUSG00000037601	Nme1	mCG145251, partial [Mus musculus]	0.609
ENSMUSG00000025499	Hras	GTPase HRas isoform X1 [Cavia porcellus]	0.608
ENSMUSG00000	Abhd17	alpha/beta hydrolase domain-containing	0.607

003346	a	protein 17A [Mus musculus]	
ENSMUSG00000062456	Rpl9-ps6	mCG130981 [Mus musculus]	0.607
ENSMUSG00000031897	Psmb10	proteasome subunit beta type-10 precursor [Mus musculus]	0.606
ENSMUSG00000042569	Dhrs7b	dehydrogenase/reductase SDR family member 7B isoform 2 [Mus musculus]	0.606
ENSMUSG00000001056	Nhp2	H/ACA ribonucleoprotein complex subunit 2 [Mus musculus]	0.606
ENSMUSG00000041736	Tspo	translocator protein [Mus musculus]	0.604
ENSMUSG00000002660	Clpp	ATP-dependent Clp protease proteolytic subunit, mitochondrial precursor [Mus musculus]	0.604
ENSMUSG00000075706	Gpx4	phospholipid hydroperoxide glutathione peroxidase, nuclear isoform B [Mus musculus]	0.604
ENSMUSG00000027613	Eif6	eukaryotic translation initiation factor 6 [Mus musculus]	0.603
ENSMUSG00000030611	Mrps11	28S ribosomal protein S11, mitochondrial isoform 1 [Mus musculus]	0.603
ENSMUSG00000	Rpl10a	60S ribosomal protein L10a [Rattus	0.602

037805		norvegicus]	
ENSMUSG00000006333	Rps9	PREDICTED: 40S ribosomal protein S9 [Marmota marmota marmota]	0.601
ENSMUSG00000028234	Rps20	PREDICTED: 40S ribosomal protein S20 [Chinchilla lanigera]	0.601
ENSMUSG00000018286	Psmb6	proteasome subunit beta type-6 precursor [Mus musculus]	0.601
ENSMUSG00000017404	Rpl19	ribosomal protein L19 [Mus musculus]	0.597
ENSMUSG00000024608	Rps14	PREDICTED: 40S ribosomal protein S14 isoform X1 [Rattus norvegicus]	0.596
ENSMUSG00000034259	Exosc4	exosome component 4, isoform CRA_b, partial [Mus musculus]	0.596
ENSMUSG00000028393	Alad	delta-aminolevulinic acid dehydratase [Mus musculus]	0.596
ENSMUSG00000074129	Rpl13a	hypothetical protein [Mus musculus]	0.593
ENSMUSG00000015943	Bola1	bolA-like protein 1 [Mus musculus]	0.592
ENSMUSG00000021773	Comtd1	catechol O-methyltransferase domain-containing protein 1 [Mus musculus]	0.592

ENSMUSG00000051557	Pusl1	tRNA pseudouridine synthase-like 1 [Mus musculus]	0.592
ENSMUSG00000049882	Vcpkmt	protein-lysine methyltransferase METTL21D [Mus musculus]	0.590
ENSMUSG00000040952	Rps19	mCG126277, isoform CRA_b [Mus musculus]	0.587
ENSMUSG00000033735	Spr	sepiapterin reductase [Mus musculus]	0.584
ENSMUSG00000041939	Mvk	mevalonate kinase isoform 1 [Mus musculus]	0.582
ENSMUSG00000004788	Eif2b2	translation initiation factor eIF-2B subunit beta [Mus musculus]	0.582
ENSMUSG00000046687	Gm5424	mCG15755 [Mus musculus]	0.582
ENSMUSG00000012848	Rps5	40S ribosomal protein S5 [Mus musculus]	0.582
ENSMUSG00000027079	Clp1	PREDICTED: polyribonucleotide 5'-hydroxyl-kinase Clp1 isoform X1 [Rattus norvegicus]	0.580
ENSMUSG00000020834	Dhrs13	dehydrogenase/reductase SDR family member 13 precursor [Mus musculus]	0.579
ENSMUSG00000	Mmp13	collagenase 3 preproprotein [Mus	0.579

050578		musculus]	
ENSMUSG00000 032288	Imp3	U3 small nucleolar ribonucleoprotein protein IMP3 [Mus musculus]	0.578
ENSMUSG00000 020826	Nos2	nitric oxide synthase, inducible isoform a [Mus musculus]	0.576
ENSMUSG00000 020153	Ndufs7	NADH dehydrogenase [ubiquinone] iron-sulfur protein 7, mitochondrial precursor [Mus musculus]	0.575
ENSMUSG00000 033020	Polr2f	DNA-directed RNA polymerases I, II, and III subunit RPABC2 [Rattus norvegicus]	0.571
ENSMUSG00000 005779	Psmb4	proteasome subunit beta type-4 precursor [Mus musculus]	0.568
ENSMUSG00000 027510	Rbm38	PREDICTED: RNA-binding protein 38 isoform X1 [Rattus norvegicus]	0.565
ENSMUSG00000 066724	Gm101 75	ATP synthase F(0) complex subunit C2, mitochondrial precursor [Mus musculus]	0.563
ENSMUSG00000 001348	Acp5	tartrate-resistant acid phosphatase type 5 precursor [Mus musculus]	0.560
ENSMUSG00000 025533	Asl	argininosuccinate lyase [Mus musculus]	0.559
ENSMUSG00000 026798	Coq4	ubiquinone biosynthesis protein COQ4 homolog, mitochondrial [Mus musculus]	0.559

ENSMUSG00000061286	Exosc5	exosome complex component RRP46 [Mus musculus]	0.554
ENSMUSG00000090137	Uba52	PREDICTED: ubiquitin-60S ribosomal protein L40 isoform X1 [Nannospalax galili]	0.553
ENSMUSG00000027679	Dnajc19	mitochondrial import inner membrane translocase subunit TIM14 isoform 2 [Mus musculus]	0.551
ENSMUSG00000019738	Polr2i	mCG22814 [Mus musculus]	0.551
ENSMUSG00000057278	Snrpg	Small nuclear ribonucleoprotein G [Cricetulus griseus]	0.542
ENSMUSG00000005481	Ddx39	ATP-dependent RNA helicase DDX39A [Mus musculus]	0.538
ENSMUSG00000066487	Gm5786	mCG20835, partial [Mus musculus]	0.532
ENSMUSG00000022157	Mcpt8	mast cell protease 8 precursor [Mus musculus]	0.532
ENSMUSG00000047721	Bola2	bolA-like protein 2 [Mus musculus]	0.529
ENSMUSG00000033220	Rac2	ras-related C3 botulinum toxin substrate 2 precursor [Mus musculus]	0.527

ENSMUSG00000076441	Ass1	argininosuccinate synthase [Mus musculus]	0.524
ENSMUSG00000062328	Rpl17	60S ribosomal protein L17 [Ictidomys tridecemlineatus]	0.510
ENSMUSG00000068240	Gm11808	PREDICTED: ubiquitin-60S ribosomal protein L40 isoform X1 [Nannospalax galili]	0.504
ENSMUSG00000005354	Txn2	thioredoxin, mitochondrial precursor [Mus musculus]	0.504
ENSMUSG00000026390	Marco	macrophage receptor MARCO [Mus musculus]	0.499
ENSMUSG00000062647	Rpl7a	Rpl7a protein, partial [Mus musculus]	0.488
ENSMUSG00000049037	Clec4a1	C-type lectin domain family 4, member a1 [Mus musculus]	0.463
ENSMUSG00000020460	Rps27a	ubiquitin-40S ribosomal protein S27a [Mus pahari]	0.448
ENSMUSG00000031722	Hp	haptoglobin isoform 1 preproprotein [Mus musculus]	0.445
ENSMUSG00000028896	Rcc1	regulator of chromosome condensation isoform 1 [Mus musculus]	0.443
ENSMUSG00000	Pgls	6-phosphogluconolactonase isoform 1	0.432

031807		[Mus musculus]	
ENSMUSG00000 027562	Car2	carbonic anhydrase 2 [Mus musculus]	0.431
ENSMUSG00000 047676	Rpsa-ps 10	mCG2650 [Mus musculus]	0.424
ENSMUSG00000 041453	Rpl21	60S ribosomal protein L21 [Mus musculus]	0.370
ENSMUSG00000 025150	Cbr2	carbonyl reductase [NADPH] 2 [Mus musculus]	0.354
